# Intermittent Fasting and Obesity-Related Health Outcomes: An Umbrella Review of Meta-analyses of Randomized Clinical Trials

**DOI:** 10.1001/jamanetworkopen.2021.39558

**Published:** 2021-12-17

**Authors:** Chanthawat Patikorn, Kiera Roubal, Sajesh K. Veettil, Viji Chandran, Tuan Pham, Yeong Yeh Lee, Edward L. Giovannucci, Krista A. Varady, Nathorn Chaiyakunapruk

**Affiliations:** 1 Department of Social and Administrative Pharmacy, Faculty of Pharmaceutical Sciences, Chulalongkorn University, Bangkok, Thailand; 2 School of Pharmacy, University of Wisconsin-Madison; 3 Department of Pharmacotherapy, College of Pharmacy, University of Utah, Salt Lake City; 4 Department of Pharmacy Practice, Manipal College of Pharmaceutical Sciences, Manipal Academy of Higher Education, Manipal, Udupi, Karnataka, India; 5 Division of Gastroenterology, Hepatology & Nutrition, Department of Internal Medicine, University of Utah, Salt Lake City; 6 School of Medical Sciences, Universiti Sains Malaysia, Kota Bharu, Malaysia; 7 Department of Epidemiology, Harvard T. H. Chan School of Public Health, Boston, Massachusetts; 8 Department of Nutrition, Harvard T. H. Chan School of Public Health, Boston, Massachusetts; 9 Department of Kinesiology and Nutrition, University of Illinois at Chicago

## Abstract

**Importance:**

Several meta-analyses of randomized clinical trials (RCTs) have demonstrated the many health benefits of intermittent fasting (IF). However, there has been little synthesis of the strength and quality of this evidence in aggregate to date.

**Objective:**

To grade the evidence from published meta-analyses of RCTs that assessed the associations of IF (zero-calorie alternate-day fasting, modified alternate-day fasting, the 5:2 diet, and time-restricted eating) with obesity-related health outcomes.

**Evidence Review:**

PubMed, Embase, and Cochrane database of systematic reviews were searched from database inception to January 12, 2021. Data analysis was conducted from April 2021 through July 2021. Meta-analyses of RCTs investigating effects of IF in adults were included. The effect sizes of IF were recalculated using a random-effects model. We assessed the quality of evidence per association by applying the GRADE criteria (Grading of Recommendations, Assessment, Development, and Evaluations) as high, moderate, low, and very low.

**Findings:**

A total of 11 meta-analyses comprising 130 RCTs (median [IQR] sample size, 38 [24-69] participants; median [IQR] follow-up period, 3 [2-5] months) were included describing 104 unique associations of different types of IF with obesity-related health outcomes (median [IQR] studies per association, 4 [3-5]). There were 28 statistically significant associations (27%) that demonstrated the beneficial outcomes for body mass index, body weight, fat mass, low-density lipoprotein cholesterol, total cholesterol, triglycerides, fasting plasma glucose, fasting insulin, homeostatic model assessment of insulin resistance, and blood pressure. IF was found to be associated with reduced fat-free mass. One significant association (1%) supported by high-quality evidence was modified alternate-day fasting for 1 to 2 months, which was associated with moderate reduction in body mass index in healthy adults and adults with overweight, obesity, or nonalcoholic fatty liver disease compared with regular diet. Six associations (6%) were supported by moderate quality evidence. The remaining associations found to be significant were supported by very low (75 associations [72%]) to low (22 associations [21%]) quality evidence.

**Conclusions and Relevance:**

In this umbrella review, we found beneficial associations of IF with anthropometric and cardiometabolic outcomes supported by moderate to high quality of evidence, which supports the role of IF, especially modified alternate-day fasting, as a weight loss approach for adults with overweight or obesity. More clinical trials with long-term follow-up are needed to investigate the effects of IF on clinical outcomes such as cardiovascular events and mortality.

## Key Points

**Question:**

What is the association of intermittent fasting with health outcomes and what is the strength of evidence of studies on intermittent fasting?

**Findings:**

This umbrella review of 11 meta-analyses of randomized clinical trials describing 104 outcomes associated with intermittent fasting on obesity-related health outcomes found 6 statistically significant associations of intermittent fasting supported by moderate to high quality of evidence. Outcomes associated with modified alternate-day fasting included a moderate reduction of body weight, body mass index, and cardiometabolic risk factors in adults with overweight or obesity.

**Meaning:**

This review suggests that intermittent fasting may have a beneficial role in improving anthropometric and cardiometabolic outcomes, especially for adults with overweight or obesity.

## Teaser

This umbrella review of meta-analyses of randomized clinical trials evaluates the association of intermittent fasting with obesity-related health outcomes.

## Introduction

Intermittent fasting (IF) has recently gained much public interest as a weight loss approach.^1^ IF is a unique dietary strategy defined as periods of eating alternated with periods of not eating (fasting).^2^ IF focuses on when food is consumed and total quantity consumed. IF works through an altered liver metabolism, referred to as the metabolic switch, where the body periodically switches from liver-derived glucose to adipose cell–derived ketones during fasting periods. Fasting stimulates adaptive cellular responses including improved glucose regulation, increased stress resistance, suppressed inflammation, and the upregulation of autophagy where damaged molecules are removed or repaired to defend against oxidative and metabolic stress.^3^ It is hypothesized that altering body metabolism will lead to long-term health benefits.^4^

Clinical trials have demonstrated the benefits of IF for many health conditions, especially obesity, diabetes, and cardiovascular diseases, through reduced weight and improved cardiometabolic parameters.^3^ Several systematic reviews and meta-analyses of randomized clinical trials (RCTs) have been published and demonstrated several health benefits of IF as well.^5,6,7,8^ However, many of these meta-analyses focused on a subset of types of IF or specific health outcomes. To date, there has been little synthesis of the strength and quality of this evidence in aggregate. This umbrella review aimed to systematically identify relevant meta-analyses of RCTs of IF, summarize their findings, and assess the strength of evidence to provide an aggregate picture of benefits associated with each type of IF on obesity-related health outcomes.

## Methods

The protocol of this study was registered with Open Science Framework (OSF)^9^ (eAppendix in the Supplement). This umbrella review reported following the 2020 Preferred Reporting Items for Systematic Reviews and Meta-analyses (PRISMA) reporting guideline.

### Search Strategy and Eligibility Criteria

We searched PubMed, EMBASE, and Cochrane database of systematic reviews from inception to January 12, 2021, to identify meta-analyses of RCTs (eTable 1 in the Supplement). No language restriction was applied. Identified articles were imported to EndNote and duplicates were removed. Two reviewers (C.P. and K.R.) independently performed screening of titles and abstracts for relevance and selected studies after examining the full text of the potentially eligible articles. Any discrepancies were resolved by discussion with the third reviewer (S.K.V.).

To be included, the studies met the following criteria: meta-analyses of RCTs investigating associations of IF with obesity-related health outcomes among adults with or without any medical conditions in comparison with any comparators including continuous energy restriction or regular diet. When more than 1 meta-analysis was available for the same research question, we selected the meta-analysis with the largest data set, as previously described.^10,11,12^ We excluded articles without full text, reviews, and meta-analyses of studies with other study designs and those without a control group.

Types of IF included in this review were (1) zero-calorie alternate-day fasting (zero-calorie ADF), which involved alternating days of fasting with zero caloric intake and days of ad libitum eating; (2) modified alternate-day fasting (MADF), which alternated between days of ad libitum eating and days of fasting with total caloric intake ranging from 0% to 40% or 0 to 600 kcal per day for 3 to 5 days per week; (3) the 5:2 diet, in which participants fasted for 1 to 2 days per week (either consecutively and nonconsecutively) with total caloric intake ranging from 0% to 40% or 0 to 600 kcal per day and 5 days of ad libitum eating; and (4) time-restricted eating (TRE), which involved fasting for 12 to 24 hours per day.^13,14^

### Data Extraction and Quality Assessment

Data extraction and quality assessment was independently performed by 2 reviewers (K.R. and V.C.) and checked by other 2 reviewers (C.P. and S.K.V.) (eMethods in the Supplement). Discrepancies were resolved with consensus. The quality of meta-analyses was assessed using A Measurement Tool to Assess Systematic Reviews (AMSTAR-2) and graded as high, moderate, low, or critically low.^15^

### Data Synthesis

Effect sizes were categorized based on the population, intervention, comparator, and outcomes to create a list of unique associations with IF. For each outcome associated with IF in a meta-analysis, we recalculated the effect sizes as mean difference (MD) with corresponding 95% CIs using the DerSimonian and Laird random-effects model.^16^ *P* < .05 was considered statistically significant in 2-sided tests. Heterogeneity was assessed with *I^2^* statistics.^17^ The evidence for small-study effects was assessed by Egger regression asymmetry test.^18^ *P* < .10 was taken as statistical evidence of the presence of small-study effects. Statistical analyses were conducted using Stata version 15.0 (StataCorp).

We assessed the quality of evidence per effect provided in a meta-analysis by applying the GRADE criteria (Grading of Recommendations, Assessment, Development, and Evaluations) in 5 domains including (1) risk of bias in the individual studies, (2) inconsistency, (3) indirectness, (4) imprecision, and (5) publication bias.^19^ We graded the strength of evidence (high, moderate, low, and very low) using GRADEpro version 3.6.1 (McMaster University).

### Sensitivity Analyses

We performed sensitivity analyses by excluding primary studies having a high risk of bias rated by the Cochrane risk-of-bias tool for RCTs and excluding small-size studies (<25th percentile) from the identified associations.^20,21^

## Results

Eleven meta-analyses were included (Figure) (justification for excluded full-text articles available in eTable 2 in the Supplement).^5,6,7,8,22,23,24,25,26,27^ Eligible meta-analyses included 130 RCTs (45 unique RCTs) with a median (IQR) sample size per RCT of 38 (24-69) studies and a follow-up period of 3 (2-5) months (Table 1). The quality of meta-analyses assessed using AMSTAR-2 found that none were rated as high confidence, 7 (64%) as moderate confidence, and 4 (36%) as low confidence (Table 1).

**Figure.**
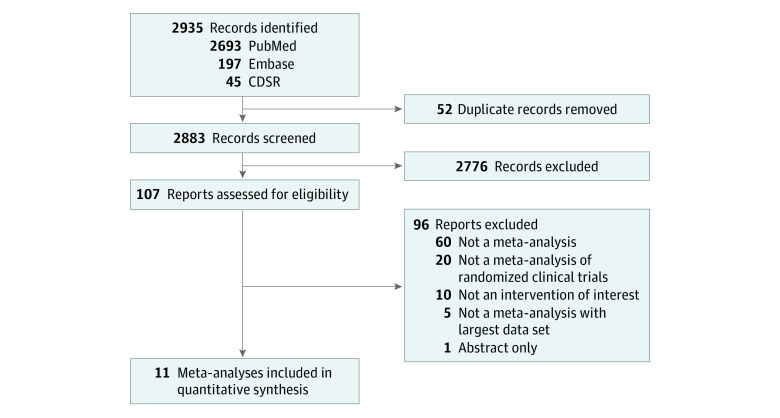
Study Selection Flow of Meta-analyses CDSR indicates Cochrane Database of Systematic Reviews.

**Table 1.** Characteristics of Meta-analyses of Randomized Clinical Trials Studying Intermittent Fasting

Source	Population	Type of IF	Comparator	Duration of fasting	No. of included studies	Total participants	Outcomes	AMSTAR-2 rating
Cioffi et al,^22^ 2018	Adults with or without medical conditions	5:2 diets, MADF	CER	2-6 mo	11	630	Body weight, fat-free mass, fat mass, HDL-C, LDL-C, TC, TG, FPG, HbA_1c_, fasting insulin, HOMA-IR, adverse events	Moderate
Harris et al,^23^ 2018	Adults with overweight or obesity	5:2 diets, MADF	RD or CER	3-6 mo	6	360	Body weight, fat-free mass, fat mass, waist circumference, HDL-C, LDL-C, TG, TC, FPG, insulin, SBP, DBP, adverse events	Moderate
Cho et al,^6^ 2019	Adults without diabetes	MADF, TRE, 0-calorie ADF	RD or CER	1-6 mo	12	545	BMI, body weight, fat-free mass, fat mass, FPG, HOMA-IR, adiponectin, leptin	Low
Roman et al,^24^ 2019	Adults with overweight or obesity	5:2 diets, MADF	CER	1-12 mo	9	782	Body weight, fat-free mass, fat mass, hip circumference, waist circumference	Low
Cui et al,^25^ 2020	Adults	MADF	RD	1-12 mo	7	269	BMI, body weight, fat-free mass, fat mass, HDL-C, LDL-C, TC, TG, FPG, HOMA-IR, SBP, DBP	Low
Meng et al,^7^ 2020	Adults	5:2 diets, MADF	RD or CER	1-12 mo	28	1528	HDL-C, LDL-C, TC, TG	Moderate
Moon et al,^5^ 2020	Adults	TRE	RD or CER	4 d to 3 mo	19	475	Body weight, fat-free mass, fat mass, HDL-C, LDL-C, TG, FPG, SBP, DBP	Moderate
Park et al,^28^ 2020	Adults	MADF	RD, CER, or TRE	1-8 mo	8	728	BMI, body weight, fat-free mass, fat mass, waist circumference, HDL-C, LDL-C, TC, TG, FPG, insulin, SBP, DBP, CRP	Moderate
Pellegrini et al,^26^ 2020	Adults who are healthy or with chronic disease not impacting outcomes	TRE	RD or CER	1-2 mo	11	452	BMI, body weight, fat-free mass, fat mass, HDL-C, LDL-C, TC, TG, FPG, fasting insulin, HOMA-IR, SBP, DBP	Low
Pureza et al,^8^ 2020	Adults with overweight or obesity	TRE	RD or TRE	1 d to 3 mo	8	264	LDL-C, HDL-C, TC, TG, FPG, fasting insulin, HOMA-IR, ghrelin	Moderate
He et al,^27^ 2021	Adults with overweight or obesity	5:2 diets, MADF	CER	3-12 mo	11	850	Body weight, fat-free mass, fat mass, waist circumference, HDL-C, LDL-C, TC, TG, FPG, HbA_1c_, fasting insulin, HOMA-IR, SBP, DBP	Moderate

Abbreviations: AMSTAR-2, A Measurement Tool to Assess Systematic Reviews; BMI, body mass index; CER, continuous energy restriction; CRP, C-reactive protein; DBP, diastolic blood pressure; FPG, fasting plasma glucose; HbA_1c_, hemoglobin A_1c_; HDL-C, high-density lipoprotein cholesterol; HOMA-IR, homeostatic model assessment for insulin resistance; LDL-C, low-density lipoprotein cholesterol; MADF, modified alternate-day fasting; RD, regular diet; SBP, systolic blood pressure; TC, total cholesterol; TG, triglyceride; TRE, time-restricted eating; ADF, alternate-day fasting.

### Description and Summary of Associations

A total of 104 unique associations were identified (Table 2; eTable 3 and eTable 4 in the Supplement). The median (IQR) number of studies per association was 4 (3-5), and the median (IQR) sample size was 266 (119-423) participants. The identified associations comprised 5 types of IF, including 2 outcomes associated with zero-calorie ADF (2%),^28^ 51 with MADF (49%),^6,7,22,25,27,28^ 28 with 5:2 diet (27%),^7,22,23,24,27^ and 23 with TRE (22%).^5,6,8,26^

**Table 2.** Summary of Significant Associations of Intermittent Fasting With Health Outcomes

Source	Outcome	Population	Duration of fasting	Type of IF	Control	No. of studies	Sample size (IF/control)	Metric	Random effect size (95% CI)	*P* value	*I^2^*, %	GRADE rating	AMSTAR-2 rating
**Anthropometric measures**													
Cui et al,^25^ 2020	BMI	Healthy adults, some with overweight, obesity, or NAFLD	1-2 mo	MADF	RD	4	82/54	MD	−1.20 (−1.44 to −0.96)	<.001	0	High	Low
Park et al,^28^ 2020	BMI	Adults with overweight or obesity	2-3 mo	MADF	RD, CER, or TRE	8	307/298	MD	−0.80 (−1.17 to −0.43)	<.001	48.4	Low	Moderate
Park et al,^28^ 2020	BMI	Adults with overweight or obesity	2-6 mo	MADF	RD, CER, or TRE	9	374/366	MD	−0.73 (−1.13 to −0.34)	.001	53.2	Very low	Moderate
He et al,^27^ 2021	Body weight, kg	Adults with overweight or obesity	2-3 mo	MADF	CER	2	39/39	MD	−1.65 (−2.73 to −0.58)	.003	0	Moderate	Moderate
He et al,^27^ 2021	Body weight, kg	Adults with overweight or obesity with no comorbidities	2-6 mo	MADF	CER	3	73/73	MD	−1.42 (−2.44 to −0.41)	.006	0	Moderate	Moderate
Park et al,^28^ 2020	Body weight, kg	Adults with overweight, some with NAFLD	1-3 mo	MADF	CER, TRE, or RD	8	307/299	MD	−1.77 (−3.19 to −0.34)	.02	55.5	Very low	Moderate
He et al,^27^ 2021	Body weight, kg	Adults with overweight or obesity, some with diabetes	3 mo	5:2 diet	RD or CER	3	117/173	MD	−1.67 (−2.79 to −0.55)	.003	0	Very low	Moderate
Pellegrini et al,^26^ 2020	Body weight, kg	Normal weight healthy male adults, some with prediabetes	1-2 mo	TRE 12-24 h	RD or CER	5	44/41	MD	−0.38 (−0.71 to −0.04)	.03	0	Low	Low
He et al,^27^ 2021	Fat-free mass, kg	Adults with obesity	2-6 mo	MADF	CER	3	73/73	MD	−0.70 (−1.38 to −0.02)	.04	0	Moderate	Moderate
Cui et al,^25^ 2020	Fat-free mass, kg	Healthy adults, some with overweight or obesity	1-12 mo	MADF	RD	5	89/73	MD	−1.38 (−2.26 to −0.49)	.002	91.0	Low	Low
Park et al,^28^ 2020	Fat mass, kg	Adults with overweight or obesity	1-2 mo	0-calorie ADF	RD or CER	2	39/39	MD	−1.99 (−2.59 to −1.38)	<.001	0	Moderate	Moderate
He et al,^27^ 2021	Fat mass, kg	Adults with obesity	2-6 mo	MADF	CER	3	73/73	MD	−1.05 (−1.98 to −0.13)	.03	0	Very low	Moderate
Park et al,^28^ 2020	Fat mass, kg	Adults with overweight or obesity, some with NAFLD	2-3 mo	MADF	RD, CER, or TRE	5	233/225	MD	−1.08 (−1.91 to −0.26)	.01	31.7	Very low	Moderate
Cui et al,^25^ 2020	Fat mass, kg	Healthy adults, some with overweight or obesity	1-12 mo	MADF	RD	6	119/107	MD	−4.96 (−8.08 to −1.85)	.002	99.0	Very low	Low
Park et al,^28^ 2020	Fat mass, kg	Adults with overweight or obesity, some with NAFLD	2-6 mo	MADF	RD, CER, or TRE	6	300/293	MD	−0.96 (−1.91 to −0.004)	.049	43.0	Very low	Moderate
Moon et al,^5^ 2020	Fat mass, kg	Adults with overweight or obesity	2-3 mo	TRE 12-21 h	RD	3	112/96	MD	−2.40 (−2.98 to −1.82)	<.001	0	Low	Moderate
**Lipid profile**													
Meng et al,^7^ 2020	LDL-C, mg/dL	Adults with normal weight, overweight, or obesity	3-12 mo	MADF	RD with exercise	5	139/140	MD	−5.14 (−7.44 to −2.83)	<.001	0	Very low	Moderate
Meng et al,^7^ 2020	LDL-C, mg/dL	Adults with normal weight, overweight, or obesity	2-12 mo	MADF	RD with exercise	7	156/154	MD	−5.23 (−7.52 to −2.94)	<.001	0	Very low	Moderate
Park et al,^28^ 2020	TC, mg/dL	Adults with overweight or obesity, some with NAFLD	2-3 mo	MADF	RD, CER, or TRE	5	250/241	MD	−10.95 (−18.98 to −2.93)	.007	2.7	Very low	Moderate
Park et al,^28^ 2020	TC, mg/dL	Adults with overweight or obesity, some with NAFLD	2-6 mo	MADF	RD or CER	6	317/309	MD	−8.13 (−15.79 to −0.46)	.04	19.9	Very low	Moderate
Meng et al,^7^ 2020	TG, mg/dL	Adults with overweight or obesity with no comorbidities	2 mo	MADF	RD or RD with exercise	2	17/14	MD	−26.84 (−52.33 to −1.35)	.04	0	Low	Moderate
Park et al,^28^ 2020	TG, mg/dL	Adults with overweight or obesity, some with NAFLD	2-3 mo	MADF	RD, CER, or TRE	5	250/241	MD	−21.67 (−39.44 to −3.89)	.02	0	Very low	Moderate
**Glycemic profile**													
Pellegrini et al,^26^ 2020	FPG, mg/dL	Healthy adults, some with overweight, obesity, or chronic diseases	1-2 mo	TRE 12-24 h	RD or CER	4	57/56	MD	−2.45 (−4.72 to −0.18)	.04	0	Very low	Low
Pureza et al,^8^ 2020	FPG, mg/dL	Adults with overweight	4 d to 3 mo	TRE 12-21 h	RD or TRE 12-15 h	7	148/147	MD	−2.75 (−4.6 to −0.91)	.003	88.7	Very low	Moderate
He et al,^27^ 2021	Fasting insulin, mIU/mL	Female adults with overweight or obesity	3-6 mo	5:2 diet	CER	2	90/94	MD	−1.00 (−1.77 to −0.39)	.002	0	Moderate	Moderate
Pureza et al,^8^ 2020	HOMA-IR	Healthy adults; some with overweight, obesity, or prediabetes	1 d to 2 mo	TRE 18 h	RD	4	59/60	MD	−0.51 (−0.82 to −0.19)	.002	50.8	Very low	Moderate
**Blood pressure**													
Cui et al,^25^ 2020	SBP, mmHg	Healthy adults, some with overweight or obesity	1-12 mo	MADF	RD	4	90/85	MD	−4.42 (−7.35 to −1.49)	.003	84.0	Very low	Low
Cui et al,^25^ 2020	DBP, mmHg	Healthy adults, some with overweight or obesity	1-12 mo	MADF	RD	4	90/85	MD	−3.41 (−5.91 to −0.92)	.003	80.0	Very low	Low

Abbreviations: AMSTAR-2, A Measurement Tool to Assess Systematic Reviews; BMI, body mass index; CER, continuous energy restriction; DBP, diastolic blood pressure; FPG, fasting plasma glucose; HOMA-IR, homeostatic model assessment of insulin resistance; IF, intermittent fasting; LDL-C, low-density lipoprotein cholesterol; MADF, modified alternate-day fasting; MD, mean difference; NAFLD, nonalcoholic fatty liver disease; RD, regular diet; SBP, systolic blood pressure; TC, total cholesterol; TG, triglycerides; TRE, time-restricted eating.

Associations analyzed included 42 (40%) anthropometric measures (ie, body mass index [BMI] [calculated as weight in kilograms divided by height in meters squared], body weight, fat-free mass, fat mass, hip circumference, and waist circumference), 34 (33%) lipid profile outcomes (ie, high-density lipoprotein cholesterol [HDL-C], low-density lipoprotein cholesterol [LDL-C], total cholesterol, and triglyceride), 15 (14%) glycemic profile outcomes (ie, fasting plasma glucose, hemoglobin A_1c_, and homeostatic model assessment of insulin resistance [HOMA-IR]), 10 (10%) blood pressure outcomes, and 1 outcome (1%) associated with C-reactive protein, adiponectin, leptin, and ghrelin levels apiece.

There were 3 associations (3%) that were specifically evaluated in healthy normal weight adults. The remaining associations were evaluated in adults with normal weight, overweight, or obesity, which in some trials included individuals with comorbidities such as diabetes (17 associations [16%]) or nonalcoholic fatty liver disease (NAFLD) (17 associations [16%]).

Strength of evidence of the 104 associations assessed using GRADE found that a majority of associations were supported by very low strength of evidence (75 associations [72%]), while the remaining associations were supported by low (22 associations [21%]), moderate (6 associations [6%]), and high level of evidence (1 association [1%]), respectively. There were 28 out of 104 associations (27%) that were statistically significant based on a random-effects model, of which 17 were supported by a very low level of evidence (61%), followed by low (5 associations [18%]), moderate (5 associations [18%]), and high (1 association [4%]) levels of evidence, respectively. These associations, which mostly involved adults with overweight or obesity, demonstrated beneficial outcomes associated with IF for BMI,^25,28^ body weight,^26,27,28^ fat mass,^5,25,27,28^ LDL-C,^7^ total cholesterol,^28^ triglyceride,^7,28^ fasting plasma glucose,^8,26^ fasting insulin,^27^ HOMA-IR,^8^ and blood pressure.^25^ IF was found to be associated with reductions in fat-free mass.^25,27^

Among the 7 associations supported by moderate to high-quality evidence, 6 were statistically significant. One association had high-quality evidence, in a meta-analysis that found MADF for 1 to 2 months was associated with reduced BMI in healthy adults and adults with overweight, obesity, or NAFLD compared with regular diet (MD, −1.20; 95% CI, −1.44 to −0.96).^25^ Five statistically significant study findings were supported by moderate quality of evidence: (1) MADF for 2 to 3 months was associated with reduced body weight in adults with overweight or obesity compared with continuous energy restriction (MD, −1.65 kg; 95% CI, −2.73 to −0.58),^27^ (2) MADF for 2 to 6 months was associated with reduced body weight in adults with obesity compared with continuous energy restriction (MD, −1.42 kg; 95% CI, −2.44 to −0.41),^27^ (3) zero-calorie ADF for 1 to 2 months was associated with reduced fat mass in adults with overweight or obesity compared with regular diet or continuous energy restriction (MD, −1.99 kg; 95% CI, −2.59 to −1.38),^28^ (4) the 5:2 diet for 3 to 6 months was associated with reduced fasting insulin in women with overweight or obesity compared with continuous energy restriction (MD, −1.00 mIU/mL; 95% CI, −1.77 to −0.39),^27^ and (5) MADF for 2 to 6 months was associated with reduced fat-free mass in adults with obesity compared with continuous energy restriction (MD, −0.70 kg; 95% CI, −1.38 to −0.02).^27^

MADF and the 5:2 diet were the only IF types associated with statistically significant weight loss in adults with overweight or obesity. In participants with obesity, body weight was found to be significantly decreased by 1.67 kg (95% CI, −2.79 to −0.55) following 3 months of the 5:2 diet. Afterwards, body weight was sustained (MD, −0.14 kg; 95% CI, −1.26 to 0.98) following 6 to 12 months.^27^ MADF was also found to be associated with improvement of several cardiometabolic risk factors in the first 2 to 12 months including LDL-C, total cholesterol, triglycerides, and blood pressure. However, small amounts of fat-free mass could be lost in the first 6 months of IF. For example, fat-free mass loss (−0.70 kg; 95% CI, −1.38 to −0.02) was associated with 2 to 6 months of MADF in adults with obesity, after which fat-free mass was sustained after 6 to 12 months of MADF (−0.01 kg; 95% CI, −0.68 to 0.69).^27^

### Sensitivity Analyses

Excluding RCTs with small size, associations initially graded as high or moderate quality retained the same rank (eTable 5 in the Supplement). When removing RCTs with a high risk of bias (measuring 7 outcomes) with very low to low quality evidence, the strength of evidence of 2 associations was upgraded to moderate. These associations were (1) the use of MADF for 1 to 3 months and the reduction of body weight in overweight adults compared with regular diet, continuous energy restriction, or TRE (MD, −2.55 kg; 95% CI, −4.43 to −0.68), and (2) the use of MADF for 3 to 12 months and reduction of LDL-C in adults compared with regular diet and exercise (MD, −3.33 mg/dL; 95% CI, −11.93 to 5.27).

## Discussion

To our knowledge, this study is the first umbrella review that systematically assessed the potential obesity-related health outcomes associated with different types of IF across a large spectrum of published meta-analyses of RCTs and evaluated the evidence by using well-recognized GRADE criteria. Our findings are important in the context of the scarcity of evidence-based support for IF that can be used to generate recommendations for clinicians and the general population. We repeated each meta-analysis with a standardized approach of random-effects analysis to allow better comparison across outcomes. We used standard approaches to assess the quality of methods of the included meta-analyses. We performed sensitivity analyses and provided additional evidence from high-quality RCTs, thus further increasing the reliability of the results.

Our findings suggest that IF is associated with successful weight loss and metabolic benefits among adults with obesity. MADF and the 5:2 diet were the only IF types that were associated with statistically significant weight loss of more than 5% in adults with overweight or obesity. In contrast, zero-calorie ADF, TRE, and RF did not. IF as a weight loss approach was found to be mostly successful in the initial phase (ie, 1-6 months), after which participants would frequently experience a plateau as additional weight loss was not further achieved because of the metabolic adaptation of the human body or decreased adherence to the assigned weight loss strategy.^29,30,31^

We identified 104 associations and found significant beneficial outcomes associated with IF on BMI, body weight, fat mass, LDL-C, total cholesterol, triglycerides, fasting plasma glucose, fasting insulin, HOMA-IR, and systolic and diastolic blood pressure, mostly in adults with overweight or obesity. IF was associated with reduced fat-free mass in adults with overweight or obesity. Only 1 effect among 28 statistically significant associations was supported by high-quality evidence in the main and sensitivity analyses, namely, the association of MADF for 1 to 2 months with reduced BMI in healthy adults and adults with overweight, obesity, or NAFLD compared with regular diet. Moderate quality of evidence also existed for an association between MADF and reduced BMI and fat-free mass in adults with overweight and/or obesity compared with continuous energy restriction. The other 2 associations had a moderate quality of evidence, namely, the use of zero-calorie ADF for the reduction of fat mass and the 5:2 diet for the reduction of fasting insulin in adults with overweight or obesity compared with continuous energy restriction and/or regular diet.

Most associations were rated as very serious in the domain of risk of bias in the individual studies according to the GRADE criteria. Associations were finally rated as high risk of bias partly because of the lack of masking participants and personnel, which could have influenced treatment allocation and outcome measurement between groups. Understandably, masking could not be appropriately performed in RCTs of IF since 2 different dietary strategies were compared.

In real-life clinical settings, IF has not been widely adopted for many reasons, including heterogeneity in techniques of IF, lack of outcome data, lack of awareness, and presumed difficulty in adherence to the regime. However, several medical conditions show promise with IF, mostly related to metabolism such as diabetes and fatty liver disease. One trial^32^ for diabetes and 2 trials^33,34^ for fatty liver disease are available. For the diabetes trial,^32^ 137 adults were randomized into either IF (2 days per week) (70 participants) or continuous energy restriction (67 participants) with outcomes of glycemic control and weight loss over 12 months. IF was found as noninferior to continuous energy restriction for both outcomes. For the fatty liver trials, both employed ADF for 8 to 12 weeks, but with Johari et al^33^ the control was normal habitual diet and with Cai et al^34^ it was time-restricted feeding. Johari et al reported reductions in BMI, alanine aminotransferase levels, steatosis and fibrosis between groups, and with Cai et al significant reductions in weight and dyslipidemia were observed with ADF. For both fatty liver trials, adherence to ADF was surprisingly good (75% to 97.5%). IF may positively affect metabolic conditions because it is associated with beneficial outcomes in anthropometric measures, as demonstrated in the current review. However, the benefits of IF are likely extending beyond weight since reduction is usually modest (ie, 2% to 10%) and thus other mechanisms are probably involved. Exact mechanisms are not entirely clear since studies are limited, but autophagy allowing liver regeneration may be important. A study suggests that peroxisome proliferator-activated receptor α activation from fasting promoted degradation of nuclear receptor co-pressor 1 and liver autophagy.^35^

To date, IF trials were mostly conducted with healthy adults and adults with overweight, obesity, or metabolic abnormalities such as diabetes and NAFLD, and were limited to surrogate anthropometric and cardiometabolic parameters with relatively short follow-up period. Therefore, more trials are needed to investigate the association of IF with: (1) a broader range of populations such as adolescents, persons older than 65 years, cancer patients, and those with metabolic derangements (eg, polycystic ovarian syndrome and thyroid disorder), (2) clinical outcomes such as liver outcomes, cancer, cardiovascular events, type 2 diabetes remission, and prevention of type 2 diabetes development from prediabetes, (3) gut microbiome and the association with improved health outcomes, and (4) short- and long-term safety outcomes such as adverse events (eg, gastrointestinal, neurological, and hematological), eating disorder syndrome, sleep parameters, reproductive hormones, fertility, and thyroid hormones. This broader scope of studies would further examine the role of IF in a clinical setting.

Our review generated several key messages that should be highly relevant to clinicians and patients, especially those who have interest in adopting the practice of IF. It is important to highlight that there is a paucity of evidence demonstrating clear and sustainable clinical benefits of IF, despite a number of mechanisms to support their benefits in both adults with comorbidities and healthy individuals.^1,3^ Another aspect that deserves attention is the lack of continuity of IF practice in these trials. This challenge will be even greater in a situation of real-world practice. Implementation research investigating strategies to facilitate the adherence of such practice is also warranted. Therefore, this lack of evidence underlines a strong need of well-designed studies to investigate long-term efficacy and safety outcomes of IF.

### Limitations

Several limitations of our study deserve discussion. First, this umbrella review focused on existing meta-analyses. We found that adverse outcomes were not included in existing meta-analyses, precluding us from making a comprehensive evaluation of both benefits and safety aspects of IF. Second, this review did not directly assess the quality of all primary studies included in each meta-analysis. Instead, we relied on the assessment reported by the study authors. Third, our review cannot answer the questions on whether IF has associations with clinical outcomes including cancer, cardiovascular events, and mortality, as these are not included in previously conducted RCTs. Moreover, we did not include meta-analyses of observational studies of IF, which may have a longer duration of follow-up. Most RCTs included in our analysis were limited to short durations of follow-up (ie, a median duration of 3 months) and relatively small number of sample size (ie, median 38 participants). This was partly because weight loss strategies made it relatively difficult for participants to adhere to the assigned treatment for a long period of time, as well as the lack of follow-up to assess the sustaining beneficial effects after cessation of IF.

## Conclusions

This umbrella review found beneficial associations of IF with anthropometric and cardiometabolic outcomes that were supported by moderate to high quality of evidence. Our results support the role of IF, especially MADF, in adults with overweight or obesity as a weight loss approach with metabolic benefits. More clinical trials with long-term follow-up are needed to investigate the effects of IF on clinical outcomes such as cardiovascular events and mortality.

## Notes

Supplement. **eAppendix.** List of Discrepancies Between the Initial Protocol and the Final Analysis
eMethods
**eTable 1.** Search Strategy From Database Inception to January 12, 2021, for Meta-Analyses of Randomized Controlled Trials **eTable 2.** Excluded Studies With Reasons From the Search for Meta-Analyses of Randomized Controlled Trials **eTable 3.** Summary of Significant Effects of Intermittent Fasting and Health Outcomes With Detail of GRADE Assessment **eTable 4.** Summary of Nonsignificant Effects of Intermittent Fasting and Health Outcomes With Detail of GRADE Assessment **eTable 5.** Summary of Sensitivity Analyses
