# Supplementary material for: Intermittent Fasting and Obesity-Related Health Outcomes: An Umbrella Review of Meta-analyses of Randomized Clinical Trials
Source: JAMA Netw Open. 2021 Dec 17;4(12):e2139558. doi: 10.1001/jamanetworkopen.2021.39558 (PMC8683964; doi:10.1001/jamanetworkopen.2021.39558)
Supplement: Supplement. — eAppendix. List of Discrepancies Between the Initial Protocol and the Final Analysis eMethods eTable 1. Search Strategy From Database Inception to January 12, 2021, for Meta-Analyses of Randomized Controlled Trials eTable 2. Excluded Studies With Reasons From the Search for Meta-Analyses of Randomized Controlled Trials eTable 3. Summary of Significant Effects of Intermittent Fasting and Health Outcomes With Detail of GRADE Assessment eTable 4. Summary of Nonsignificant Effects of Intermittent Fasting and Health Outcomes With Detail of GRADE Assessment eTable 5. Summary of Sensitivity Analyses [file jamanetwopen-e2139558-s001.pdf]

Supplemental Online Content

Patikorn C, Roubal K, Veettil SK, et al. Intermittent fasting and obesity-related health outcomes. *JAMA Netw Open.* 2021;4(12):e2139558.
doi:10.1001/jamanetworkopen.2021.39558

eAppendix. List of Discrepancies Between the Initial Protocol and the Final Analysis

eMethods

eTable 1. Search Strategy From Database Inception to January 12, 2021, for Meta-Analyses of Randomized Controlled Trials

eTable 2. Excluded Studies With Reasons From the Search for Meta-Analyses of Randomized Controlled Trials

eTable 3. Summary of Significant Effects of Intermittent Fasting and Health Outcomes With Detail of GRADE Assessment

eTable 4. Summary of Nonsignificant Effects of Intermittent Fasting and Health Outcomes With Detail of GRADE Assessment

eTable 5. Summary of Sensitivity Analyses

This supplemental material has been provided by the authors to give readers additional information about their work.

© 2021 Patikorn C et al. *JAMA Network Open.*

eAppendix. List of Discrepancies Between the Initial Protocol and the Final Analysis

Initial protocol	Differences	Rationale
Types of intermittent fasting included in this review were (1) zero-calorie alternate-day fasting (0-calorie ADF) – alternating between days of fasting with zero caloric intake and days of ad libitum eating, (2) modified alternate-day fasting (MADF) – alternating between days of ad libitum eating and days of fasting with total caloric intake ranging from 0-40% or 0-600 kcal per day for 3-5 days per week, (3) the 5:2 diet – fasting for 1-2 consecutive or non-consecutive days per week with total caloric intake ranging from 0-40% or 0-600 kcal per day and 5 days of ad libitum eating, and (4) time-restricted eating (TRE) – fasting for 12 to 24 hours per day, which also includes Ramadan fasting (RF) – fasting from sunrise to sunset with fasting time of 9 to 22 hours per day.	We have removed Ramadan fasting from the eligible types of intermittent fasting in this review.	Ramadan fasting was considered not comparable with other types of intermittent fasting especially when individuals could not drink during fasting and the food eaten after fasting is generally not representative of what they usually eat.
Additional search was also performed in PubMed, EMBASE, and CENTRAL from 1 January to 30 April 2021 to identify recently published randomized controlled trial to update existing meta-analyses.	We have removed search for recently published randomized controlled trial of intermittent fasting.	Umbrella review should focus on meta-analyses as the unit of analysis, not individual randomized controlled trials.

© 2021 Patikorn C et al. *JAMA Network Open.*

eMethods

Data extraction

A data extraction sheet was developed and pilot-tested on five randomly selected included articles and then refined until finalization. Two reviewers (K.R. and V.C.) independently performed data extraction. The extracted data were divided and checked by other two reviewers (C.P. and S.K.V). Discrepancies were resolved with consensus. The corresponding authors of the included articles were contacted when the data provided in the published articles were insufficient for evidence synthesis.

The following data were extracted from the eligible articles: name of the first author, year of publication, characteristics of population, type of intermittent fasting, type of comparator, duration of follow-up, duration of fasting, number of included studies, number of participants in each arm of the trial, outcomes, study-specific effect sizes (i.e., mean difference or weighted mean difference) and their corresponding confidence intervals and *P* values.

© 2021 Patikorn C et al. *JAMA Network Open.*

eTable 1. Search Strategy From Database Inception to January 12, 2021, for Meta-Analyses of Randomized Controlled Trials

Database	Search term	Results
PubMed	('intermittent fasting' OR 'intermittent energy restriction' OR 'periodic fasting' OR 'alternate-day fasting' OR 'alternate day fasting' OR 'modified alternate-day fasting' OR 'modified alternate day fasting' OR 'time-restricted feeding' OR 'time restricted feeding' OR 'whole day fasting' OR ramadan OR ramadhan) AND ('systematic review' OR 'systematic literature review' OR 'meta-analysis' OR 'meta-analyses' OR 'meta analysis' OR 'meta analyses')	2693
EMBASE	('intermittent fasting' OR 'intermittent energy restriction' OR 'periodic fasting' OR 'alternate-day fasting' OR 'alternate day fasting' OR 'modified alternate-day fasting' OR 'modified alternate day fasting' OR 'time-restricted feeding' OR 'time restricted feeding' OR 'whole day fasting' OR ramadan OR ramadhan) AND ('systematic review' OR 'systematic literature review' OR 'meta-analysis' OR 'meta-analyses' OR 'meta analysis' OR 'meta analyses')	197
Cochrane Database of Systematic Review	('intermittent fasting' OR 'intermittent energy restriction' OR 'periodic fasting' OR 'alternate-day fasting' OR 'alternate day fasting' OR 'modified alternate-day fasting' OR 'modified alternate day fasting' OR 'time-restricted feeding' OR 'time restricted feeding' OR 'whole day fasting' OR ramadan OR ramadhan) AND ('systematic review' OR 'systematic literature review' OR 'meta-analysis' OR 'meta-analyses' OR 'meta analysis' OR 'meta analyses') in Title Abstract Keyword - in Cochrane Reviews	45
**TOTAL**		**2935**

© 2021 Patikorn C et al. *JAMA Network Open.*

eTable 2. Excluded Studies With Reasons From the Search for Meta-Analyses of Randomized Controlled Trials

Reasons for exclusion	References
Not a meta-analysis (n = 60)	1. Müller H, de Toledo FW, Resch KL. Fasting followed by vegetarian diet in patients with rheumatoid arthritis: a systematic review. Scand J Rheumatol. 2001;30(1):1-10. doi:10.1080/030097401750065256<br>2. Azizi F. Islamic fasting and health. Ann Nutr Metab. 2010;56(4):273-82. doi:10.1159/000295848<br>3. Horne BD, Muhlestein JB, May HT, et al. Relation of routine, periodic fasting to risk of diabetes mellitus, and coronary artery disease in patients undergoing coronary angiography. Article. American Journal of Cardiology. 2012;109(11):1558-1562. doi:10.1016/j.amjcard.2012.01.379<br>4. Sadeghpour S, Keshteli AH, Daneshpajouhnejad P, Jahangiri P, Adibi P. Ramadan fasting and digestive disorders: SEPAHAN systematic review no. 7. Article. Journal of Research in Medical Sciences. 2012;17(1 SPL.1):S150-S158.<br>5. Fond G, Macgregor A, Leboyer M, Michalsen A. Fasting in mood disorders: Neurobiology and effectiveness. A review of the literature. Review. Psychiatry Research. 2013;209(3):253-258. doi:10.1016/j.psychres.2012.12.018<br>6. Salim I, Al Suwaidi J, Ghadban W, Alkilani H, Salam AM. Impact of religious Ramadan fasting on cardiovascular disease: A systematic review of the literature. Review. Current Medical Research and Opinion. 2013;29(4):343-354. doi:10.1185/03007995.2013.774270<br>7. Bragazzi NL. Ramadan fasting and chronic kidney disease: A systematic review. Review. Journal of Research in Medical Sciences. 2014;19(7):665-676.

© 2021 Patikorn C et al. *JAMA Network Open.*

	8. Hankir A, Hankir M, Zaman R. Should Ramadan be prescribed after Christmas? Obesity in the healthcare profession and the health benefits of fasting. Article. BMJ Case Reports. 2014;doi:10.1136/bcr-2013-202704<br>9. Hassan A, Meo SA. Diabetes during Ramadan: Underestimated, under-investigated, needs more attention. Article. European Review for Medical and Pharmacological Sciences. 2014;18(22):3528-3533.<br>10. Javadi MA, Assadi M, Einollahi B, Rabei HM, Afarid M. The effects of ramadan fasting on the health and function of the eye. Article. Journal of Research in Medical Sciences. 2014;19(8):786-791.<br>11. Rouhani MH, Azadbakht L. Is Ramadan fasting related to health outcomes? A review on the related evidence. Article. Journal of Research in Medical Sciences. 2014;19(10):987-992.<br>12. Saeidifard F, Soltani A. Is scientific production problem-oriented? Diabetes and Ramadan as an example. Letter. Oman Medical Journal. 2014;29(3):244. doi:10.5001/omj.2014.62<br>13. Bragazzi NL, Briki W, Khabbache H, et al. Ramadan fasting and infectious diseases: A systematic review. Review. Journal of Infection in Developing Countries. 2015;9(11):1186-1194. doi:10.3855/jidc.5815<br>14. Ells LJ, Atkinson G, McGowan VJ, Hamilton S, Waller G, Harrison S. Intermittent fasting interventions for the treatment of overweight and obesity in adults aged 18 years and over: A systematic review protocol. Article. JBI Database of Systematic Reviews and Implementation Reports. 2015;13(10):60-68. doi:10.11124/jbisrir-2015-2363<br>15. Fenneni MA, Latiri I, Aloui A, Rouatbi S, Chamari K, Saad HB. Critical analysis of the published literature about the effects of Ramadan intermittent fasting on healthy children's physical capacities. Article. Libyan Journal of Medicine. 2015;10doi:10.3402/ljm.v10.28351

© 2021 Patikorn C et al. *JAMA Network Open.*

	16. Hankey C, Klukowska D, Lean M. A systematic review of the literature on intermittent fasting for weight management. Conference Abstract. FASEB Journal. 2015;29(1)<br>17. Horne BD, Muhlestein JB, Anderson JL. Health effects of intermittent fasting: hormesis or harm? A systematic review. Am J Clin Nutr. Aug 2015;102(2):464-70. doi:10.3945/ajcn.115.109553<br>18. Mazidi M, Rezaie P, Chaudhri O, Karimi E, Nematy M. The effect of Ramadan fasting on cardiometabolic risk factors and anthropometrics parameters: A systematic review. Review. *Pakistan Journal of Medical Sciences*. 2015;31(5)<br>19. Seimon RV, Roekenes JA, Zibellini J, et al. Do intermittent diets provide physiological benefits over continuous diets for weight loss? A systematic review of clinical trials. *Mol Cell Endocrinol*. Dec 15 2015;418 Pt 2:153-72. doi:10.1016/j.mce.2015.09.014<br>20. Bragazzi NL, Briki W, Khabbache H, et al. Ramadan fasting and patients with cancer: State-of-the-art and future prospects. Article. *Frontiers in Oncology*. 2016;6(FEB)doi:10.3389/fonc.2016.00027<br>21. Adawi M, Watad A, Brown S, et al. Ramadan fasting exerts immunomodulatory effects: Insights from a systematic review. Review. *Frontiers in Immunology*. 2017;8(NOV)doi:10.3389/fimmu.2017.01144<br>22. Ochoa Rivera T, Lopez-Teros M, Escalante-Izeta E, Gamboa-Melendez MA, Tello-Casillas JK, Méndez-Montoya AF. Fasting, intermittent fasting or caloric restriction as nutritional management of adults with type 2 diabetes. A systematic review (preliminary results). Conference Abstract. *Annals of Nutrition and Metabolism*. 2017;71:958. doi:10.1159/000480486<br>23. Patterson RE, Sears DD. Metabolic Effects of Intermittent Fasting. 2017. p. 371-393.<br>24. Qasrawi SO, Pandi-Perumal SR, BaHammam AS. The effect of intermittent fasting during Ramadan on sleep, sleepiness, cognitive function, and circadian rhythm. Review. *Sleep and Breathing*. 2017;21(3):577-586. doi:10.1007/s11325-017-1473-x

© 2021 Patikorn C et al. *JAMA Network Open.*

	25. Tello Casillas JK, Lopez-Teros M, Ochoa Rivera T, Escalante-Izeta E, Gamboa-Melendez MA. Fasting and caloric restriction in elderly with cardiovascular disease: A review. Conference Abstract. *Annals of Nutrition and Metabolism*. 2017;71:952. doi:10.1159/000480486<br>26. Lee SWH, Lai NM, Chen WS, Sellappans R. Interventions for people with type 2 diabetes mellitus fasting during Ramadan. Review. *Cochrane Database of Systematic Reviews*. 2018;2018(11)doi:10.1002/14651858.CD013178<br>27. Allaf M, Elghazaly H, Mohamed OG, et al. Intermittent fasting for the prevention of cardiovascular disease. Article. *Cochrane Database of Systematic Reviews*. 2019;2019(11)doi:10.1002/14651858.CD013496<br>28. Aloui A, Baklouti H, Souissi N, Chtourou H. Effects of ramadan fasting on body composition in athletes: A systematic review. Article. *Tunisie Medicale*. 2019;97(10):1087-1094.<br>29. Bello AK, Kurzawa J, Osman MA, et al. Impact of Ramadan fasting on kidney function and related outcomes in patients with chronic kidney disease: A systematic review protocol. Review. *BMJ Open*. 2019;9(8)doi:10.1136/bmjopen-2018-022710<br>30. Chtourou H, Trabelsi K, Boukhris O, Ammar A, Shephard RJ, Bragazzi NL. Effects of ramadan fasting on physical performances in soccer players: A systematic review. Article. *Tunisie Medicale*. 2019;97(10):1114-1131.<br>31. Home BD, Bartholomew C, Anderson JL, et al. Intermittent fasting lifestyle and human longevity in cardiac catheterization populations. Conference Abstract. *Circulation*. 2019;140doi:10.1161/circ.140.suppl_1.11123<br>32. Ismail S, Manaf R, Mahmud A. Comparison of time-restricted feeding and islamic fasting: A scoping review. Article. *Eastern Mediterranean Health Journal*. 2019;25(4):239-245. doi:10.26719/emhj.19.011<br>33. Lessan N, Ali T. Energy metabolism and intermittent fasting: The ramadan perspective. Review. Nutrients. 2019;11(5)doi:10.3390/nu11051192

© 2021 Patikorn C et al. *JAMA Network Open.*

34. Nicolini D, Concina D, Farsoni M, Sarro A, Rinaldi C, Panella M. The effects of fasting regimens on health-results of a systematic overview. Conference Abstract. Journal of Preventive Medicine and Hygiene. 2019;60(3):E155. doi:10.15167/2421-4248/jpmh2019.60.3s1
35. Philippou E, Petersson SD, Erodotou S, Giallouri E, Rodomar C, Nikiphorou E. Dietary intake, dietary interventions, nutrient supplements and rheumatoid arthritis: Systematic review of the evidence. Conference Abstract. *Proceedings of the Nutrition Society*. 2019;78(OCE1)doi:10.1017/S0029665119000259
36. Pourabbasi A, Ebrahimnegad Shirvani MS, Shams AH. Does Islamic fasting affect cognitive functions in adolescents? A systematic review. Article. *JPMA The Journal of the Pakistan Medical Association*. 2019;69(8):1164-1169.
37. Rashid F, Abdelgadir E. A systematic review on efficacy and safety of the current hypoglycemic agents in patients with diabetes during Ramadan fasting. Article. *Diabetes and Metabolic Syndrome: Clinical Research and Reviews*. 2019;13(2):1413-1429. doi:10.1016/j.dsx.2019.02.005
38. Rynders CA, Thomas EA, Zaman A, Pan Z, Catenacci VA, Melanson EL. Effectiveness of intermittent fasting and time-restricted feeding compared to continuous energy restriction for weight loss. Review. *Nutrients*. 2019;11(10)doi:10.3390/nu11102442
39. Trabelsi K, Ammar A, Zlitni S, et al. Practical recommendations to improve sleep during ramadan observance in healthy practitioners of physical activity. Article. *Tunisie Medicale*. 2019;97(10):1077-1086.
40. Trabelsi K, Shephard RJ, Boukhris O, et al. Effects of ramadan fasting on athletes' hematological indices: A systematic review. Article. *Tunisie Medicale*. 2019;97(10):1104-1113.
41. Zoukal S, Hassoune S. The effects of ramadan fasting during pregnancy on fetal development: A general review. Article. *Tunisie Medicale*. 2019;97(10):1132-1138.

© 2021 Patikorn C et al. *JAMA Network Open.*

	42. Almulhem M, Susarla R, Alabdulaali L, et al. The effect of Ramadan fasting on cardiovascular events and risk factors in patients with type 2 diabetes: A systematic review. Review. *Diabetes Research and Clinical Practice*. 2020;159doi:10.1016/j.diabres.2019.107918
	43. Amjadi M, Soleimanzadeh F, Ghamatzadeh H, Hajebrahimi S, Hosseinifard H, Salehi Pourmehr H. Ramadan Fasting and Kidney Stones: A Systematic Review. Article in Press. *Urology journal*. 2020;doi:10.22037/uj.v16i7.6373
	44. Beshyah SA, Ali KF, Hafidh K, Hajjaji IM. Ramadan Fasting and Diabetes 2019: The Year in Review. Article in Press. *Diabetes research and clinical practice*. 2020:108593. doi:10.1016/j.diabres.2020.108593
	45. Faris MAIE, Salem ML, Jahrami HA, Madkour MI, Ba Hammam AS. Ramadan intermittent fasting and immunity: An important topic in the era of COVID-19. Review. *Annals of Thoracic Medicine*. 2020;15(3):125-133. doi:10.4103/atm.ATM_151_20
	46. Husain S, Zafar M, Ullah R. Ramadan and public health: A bibliometric analysis of top cited articles from 2004 to 2019. Article. *Journal of Infection and Public Health*. 2020;13(2):275-280. doi:10.1016/j.jiph.2019.11.006
	47. Igwe O, Sone M, Matveychuk D, Baker GB, Dursun SM. A review of effects of calorie restriction and fasting with potential relevance to depression. Article in Press. *Progress in Neuro-Psychopharmacology and Biological Psychiatry*. 2020;doi:10.1016/j.pnpbp.2020.110206
	48. Keenan S, Cooke MB, Belski R. The effects of intermittent fasting combined with resistance training on lean body mass: A systematic review of human studies. Review. *Nutrients*. 2020;12(8):1-17. doi:10.3390/nu12082349
	49. Kirubarajan A, Lam ACL, Khan S, Yau M, Golda N, Buckley R. The association between renal stones and fasting: A systematic review. Review. *Canadian Urological Association Journal*. 2020;15(3)doi:10.5489/CUAJ.6664

© 2021 Patikorn C et al. *JAMA Network Open.*

	50. Lima CHR, Oliveira IKF, de Macêdo Gonçalves Frota K, et al. Impact of intermittent fasting on body weight in overweight and obese individuals. Review. *Revista da Associacao Medica Brasileira*. 2020;66(2):222-226. doi:10.1590/1806-9282.66.2.222<br>51. Lutes C, Zelig R, Rigassio Radler D. Safety and Feasibility of Intermittent Fasting during Chemotherapy for Breast Cancer: A Review of the Literature. Review. *Topics in Clinical Nutrition*. 2020;35(2):168-177. doi:10.1097/TIN.0000000000000215<br>52. Mohd Yusof BN, Yahya NF, Hasbullah FY, et al. Ramadan-focused nutrition therapy for people with diabetes: A narrative review. Article in Press. *Diabetes Research and Clinical Practice*. 2020;doi:10.1016/j.diabres.2020.108530<br>53. Nasaruddin ML, Syed Abd Halim SA, Kamaruzzaman MA. Studying the relationship of intermittent fasting and β-amyloid in animal model of alzheimer's disease: A scoping review. Review. *Nutrients*. 2020;12(10):1-13. doi:10.3390/nu12103215<br>54. Rashid F, Abdelgadir E, Bashier A. A systematic review on the safety of Ramadan fasting in high-risk patients with Diabetes. Article. *Diabetes Research and Clinical Practice*. 2020;164doi:10.1016/j.diabres.2020.108161<br>55. Réda A, Wassil M, Mériem M, et al. Food timing, circadian rhythm and chrononutrition: A systematic review of time-restricted eating's effects on human health. Review. *Nutrients*. 2020;12(12):1-15. doi:10.3390/nu12123770<br>56. Santos HO, Genario R, Macedo RCO, Pareek M, Tinsley GM. Association of breakfast skipping with cardiovascular outcomes and cardiometabolic risk factors: an updated review of clinical evidence. Article in Press. *Critical reviews in food science and nutrition*. 2020:1-9. doi:10.1080/10408398.2020.1819768<br>57. Sarro A, Payedimarri AB, Concina D, Farsoni M, Piu N, Panella M. The efficacy of fasting regimens on health outcomes: a systematic overview. Article in Press. *Minerva gastroenterologica e dietologica*. 2020;doi:10.23736/S1121-421X.20.02757-9

© 2021 Patikorn C et al. *JAMA Network Open.*

	58. Vitale R, Kim Y. The Effects of Intermittent Fasting on Glycemic Control and Body Composition in Adults with Obesity and Type 2 Diabetes: A Systematic Review. Review. *Metabolic Syndrome and Related Disorders*. 2020;18(10):450-461. doi:10.1089/met.2020.0048<br>59. Welton S, Minty R, O'Driscoll T, et al. Intermittent fasting and weight loss Systematic review. Review. *Canadian Family Physician*. 2020;66(2):117-125.<br>60. Zorbas C, Reeve E, Naughton S, et al. The Relationship Between Feasting Periods and Weight Gain: a Systematic Scoping Review. Review. *Current Obesity Reports*. 2020;9(1):39-62. doi:10.1007/s13679-020-00370-5
Not a meta-analysis of randomized controlled trials (n = 20)	1. Kul S, Savaş E, Öztürk ZA, Karadağ G. Does Ramadan fasting alter body weight and blood lipids and fasting blood glucose in a healthy population? A meta-analysis. *J Relig Health*. Jun 2014;53(3):929-42. doi:10.1007/s10943-013-9687-0<br>2. Sadeghirad B, Motaghipisheh S, Kolahdooz F, Zahedi MJ, Haghdoost AA. Islamic fasting and weight loss: a systematic review and meta-analysis. *Public Health Nutr*. Feb 2014;17(2):396-406. doi:10.1017/s1368980012005046<br>3. Bragazzi NL. Ramadan fasting and chronic kidney disease: Does estimated glomerular filtration rate change after and before Ramadan? Insights from a mini meta-analysis. Article. *International Journal of Nephrology and Renovascular Disease*. 2015;8:53-57. doi:10.2147/IJNRD.S61718<br>4. Turin TC, Ahmed S, Shommu NS, et al. Ramadan fasting is not usually associated with the risk of cardiovascular events: A systematic review and meta-analysis. J Family Community Med. May-Aug 2016;23(2):73-81. doi:10.4103/2230-8229.181006<br>5. Glazier JD, Hayes DJL, Hussain S, et al. The effect of Ramadan fasting during pregnancy on perinatal outcomes: a systematic review and meta-analysis. *BMC Pregnancy Childbirth*. Oct 25 2018;18(1):421. doi:10.1186/s12884-018-2048-y

© 2021 Patikorn C et al. *JAMA Network Open.*

	6. Aydin N, Kul S, Karadağ G, Tabur S, Araz M. Effect of Ramadan fasting on glycaemic parameters & body mass index in type II diabetic patients: A meta-analysis. *Indian J Med Res*. Dec 2019;150(6):546-556. doi:10.4103/ijmr.IJMR_1380_17<br>7. Binsalih S, Al Sayyari RA, Sheikho M, Hejaili FF, Al Sayyari AA. Effect of Fasting the Whole Month of Ramadan on Renal Function Among Muslim Patients With Kidney Transplant: A Meta-Analysis. *Exp Clin Transplant*. Oct 2019;17(5):588-593. doi:10.6002/ect.2019.0245<br>8. Faris MAIE, Jahrami HA, Obaideen AA, Madkour MI. Impact of diurnal intermittent fasting during Ramadan on inflammatory and oxidative stress markers in healthy people: Systematic review and meta-analysis. Review. *Journal of Nutrition and Intermediary Metabolism*. 2019;15:18-26. doi:10.1016/j.jnim.2018.11.005<br>9. Fernando HA, Zibellini J, Harris RA, Seimon RV, Sainsbury A. Effect of Ramadan Fasting on Weight and Body Composition in Healthy Non-Athlete Adults: A Systematic Review and Meta-Analysis. Nutrients. Feb 24 2019;11(2)doi:10.3390/nu11020478<br>10. Mirmiran P, Bahadoran Z, Gaeini Z, Moslehi N, Azizi F. Effects of Ramadan intermittent fasting on lipid and lipoprotein parameters: An updated meta-analysis. *Nutr Metab Cardiovasc Dis*. Sep 2019;29(9):906-915. doi:10.1016/j.numecd.2019.05.056<br>11. Abaïdia AE, Daab W, Bouzid MA. Effects of Ramadan Fasting on Physical Performance: A Systematic Review with Meta-analysis. Review. *Sports medicine (Auckland, NZ)*. 2020;50(5):1009-1026. doi:10.1007/s40279-020-01257-0<br>12. Al-Islam Faris M, Jahrami H, BaHammam A, Kalaji Z, Madkour M, Hassanein M. A systematic review, meta-analysis, and meta-regression of the impact of diurnal intermittent fasting during Ramadan on glucometabolic markers in healthy subjects. Article in Press. *Diabetes research and clinical practice*. 2020:108226. doi:10.1016/j.diabres.2020.108226

© 2021 Patikorn C et al. *JAMA Network Open.*

	13. Correia JM, Santos I, Pezarat-Correia P, Minderico C, Mendonca GV. Effects of intermittent fasting on specific exercise performance outcomes: A systematic review including meta-analysis. Review. Nutrients. 2020;12(5)doi:10.3390/nu12051390<br>14. Faris MAIE, Alsibai J, Jahrami HA, Obaideen AA, Jahrami HA, Obaideen AA. Impact of Ramadan diurnal intermittent fasting on the metabolic syndrome components in healthy, non-athletic Muslim people aged over 15 years: A systematic review and meta-analysis. Review. *British Journal of Nutrition*. 2020;123(1):1-22. doi:10.1017/S000711451900254X<br>15. Faris MAIE, Jahrami HA, Alhayki FA, et al. Effect of diurnal fasting on sleep during Ramadan: a systematic review and meta-analysis. Review. *Sleep and Breathing*. 2020;24(2):771-782. doi:10.1007/s11325-019-01986-1<br>16. Jahrami HA, Alsibai J, Clark CCT, Faris MAIE. A systematic review, meta-analysis, and meta-regression of the impact of diurnal intermittent fasting during Ramadan on body weight in healthy subjects aged 16 years and above. Review. *European Journal of Nutrition*. 2020;59(6):2291-2316. doi:10.1007/s00394-020-02216-1<br>17. Kord-Varkaneh H, Nazary-Vannani A, Mokhtari Z, et al. The Influence of Fasting and Energy Restricting Diets on Blood Pressure in Humans: A Systematic Review and Meta-Analysis. *High Blood Press Cardiovasc Prev*. Aug 2020;27(4):271-280. doi:10.1007/s40292-020-00391-0<br>18. Tahapary DL, Astrella C, Kristanti M, Harbuwono DS, Soewondo P. The impact of Ramadan fasting on metabolic profile among type 2 diabetes mellitus patients: A meta-analysis. Review. *Diabetes and Metabolic Syndrome: Clinical Research and Reviews*. 2020;14(5):1559-1570. doi:10.1016/j.dsx.2020.07.033<br>19. Trabelsi K, Ammar A, Boukhris O, et al. Effects of ramadan observance on dietary intake and body composition of adolescent athletes: Systematic review and meta-analysis. Review. *Nutrients*. 2020;12(6)doi:10.3390/nu12061574

© 2021 Patikorn C et al. *JAMA Network Open.*

	20. Trabelsi K, Bragazzi N, Zlitni S, et al. Observing Ramadan and sleep-wake patterns in athletes: a systematic review, meta-analysis and meta-regression. *Br J Sports Med*. Jun 2020;54(11):674-680. doi:10.1136/bjsports-2018-099898
Not intervention of interest (n = 10)	1. Avenell A, Brown TJ, McGee MA, et al. What are the long-term benefits of weight reducing diets in adults? A systematic review of randomized controlled trials. J Hum Nutr Diet. Aug 2004;17(4):317-35. doi:10.1111/j.1365-277X.2004.00531.x<br>2. Gray LJ, Dales J, Brady EM, Khunti K, Hanif W, Davies MJ. The safety and effectiveness of non-insulin glucose lowering agents in the treatment of people with Type 2 diabetes who observe Ramadan: A systematic review and metaanalysis. Conference Abstract. *Diabetic Medicine*. 2015;32:203. doi:10.1111/dme.12668_1<br>3. Mbanya JC, Al-Sifri S, Abdel-Rahim A, Satman I. Incidence of hypoglycemia in patients with type 2 diabetes treated with gliclazide versus DPP-4 inhibitors during Ramadan: A meta-analytical approach. *Diabetes Res Clin Pract*. Aug 2015;109(2):226-32. doi:10.1016/j.diabres.2015.04.030<br>4. Lee SWH, Lee JY, Tan CSS, Wong CP. Strategies to Make Ramadan Fasting Safer in Type 2 Diabetics: A Systematic Review and Network Meta-analysis of Randomized Controlled Trials and Observational Studies. *Medicine (Baltimore)*. Jan 2016;95(2):e2457. doi:10.1097/md.0000000000002457<br>5. Loh HH, Yee A, Loh HS, Sukor N, Kamaruddin NA. Comparative studies of dipeptidyl peptidase 4 inhibitor vs sulphonylurea among Muslim Type 2 diabetes patients who fast in the month of Ramadan: A systematic review and meta-analysis. *Prim Care Diabetes*. Jun 2016;10(3):210-9. doi:10.1016/j.pcd.2015.09.001<br>6. Loh HH, Lim LL, Loh HS, Yee A. Safety of Ramadan fasting in young patients with type 1 diabetes: A systematic review and meta-analysis. *J Diabetes Investig*. Nov 2019;10(6):1490-1501. doi:10.1111/jdi.13054

© 2021 Patikorn C et al. *JAMA Network Open.*

	7. Borgundvaag E, Mak J, Kramer CK. Metabolic impact of intermittent fasting in patients with type 2 diabetes mellitus: a systematic review and meta-analysis of interventional studies. Article in Press. *The Journal of clinical endocrinology and metabolism*. 2020;doi:10.1210/clinem/dgaa926<br>8. Enríquez Guerrero A, San Mauro Martín I, Garicano Vilar E, Camina Martín MA. Effectiveness of an intermittent fasting diet versus continuous energy restriction on anthropometric measurements, body composition and lipid profile in overweight and obese adults: a meta-analysis. Article in Press. *European Journal of Clinical Nutrition*. 2020;doi:10.1038/s41430-020-00821-1<br>9. Fatahi S, Nazary-Vannani A, Sohouli MH, et al. The effect of fasting and energy restricting diets on markers of glucose and insulin controls: a systematic review and meta-analysis of randomized controlled trials. *Critical Reviews in Food Science and Nutrition*. Aug 3 2020:1-12. doi:10.1080/10408398.2020.1798350<br>10. Rahmani J, Kord Varkaneh H, Clark C, et al. The influence of fasting and energy restricting diets on IGF-1 levels in humans: A systematic review and meta-analysis. *Ageing Res Rev*. Aug 2019;53:100910. doi:10.1016/j.arr.2019.100910
Not a meta-analysis with the largest data set (n = 5)	1. Alhamdan BA, Garcia-Alvarez A, Alzahrnai AH, et al. Alternate-day versus daily energy restriction diets: which is more effective for weight loss? A systematic review and meta-analysis. Review. Obesity Science and Practice. 2016;2(3):293-302. doi:10.1002/osp4.52<br>2. Headland M, Clifton PM, Carter S, Keogh JB. Weight-Loss Outcomes: A Systematic Review and Meta-Analysis of Intermittent Energy Restriction Trials Lasting a Minimum of 6 Months. *Nutrients*. Jun 8 2016;8(6)doi:10.3390/nu8060354<br>3. Harris L, McGarty A, Hutchison L, Ells L, Hankey C. Short-term intermittent energy restriction interventions for weight management: a systematic review and meta-analysis. *Obes Rev*. Jan 2018;19(1):1-13. doi:10.1111/obr.12593

© 2021 Patikorn C et al. *JAMA Network Open.*

	4. Yan S, Wang C, Zhao H, et al. Effects of fasting intervention regulating anthropometric and metabolic parameters in subjects with overweight or obesity: a systematic review and meta-analysis. *Food Funct*. May 1 2020;11(5):3781-3799. doi:10.1039/d0fo00287a<br>5. Wang X, Yan Q, Liao Q, et al. Effects of intermittent fasting diets on plasma concentrations of inflammatory biomarkers: A systematic review and meta-analysis of randomized controlled trials. Review. *Nutrition*. 2020;79-80doi:10.1016/j.nut.2020.110974
Abstract only<br>(n = 1)	1. Francis L, Young J, Lara J. The impact of intermittent fasting on body composition and cardiovascular biomarkers: A systematic review and meta-analysis. Conference Abstract. *Proceedings of the Nutrition Society*. 2017;76(OCE2):E42. doi:10.1017/S0029665117000982

© 2021 Patikorn C et al. *JAMA Network Open.*

eTable 3. Summary of Significant Effects of Intermittent Fasting and Health Outcomes With Detail of GRADE Assessment

Source	Outcome	Population	Duration of fasting	Type of IF	Control (C)	No. of studies	Sample size (IF/C)	Metric	Random effect size (95% CI)	*P* value	GRADE evidence (Not serious (NS), serious (S), very serious (VS))						AMSTAR-2
											Risk of bias	Inconsistency, I^2, %	Indirectness	Imprecision	publication bias,	Overall certainty of evidence	
Anthropometric measures																	
Cui et al,[13] 2020	BMI, kg/m^2	Healthy adults, some with overweight, obesity, or NAFLD	1-2 months	MADF	RD	4	82/54	MD	-1.20 (-1.44 to -0.96)	1 x 10^{-5}	NS	NS, 0	NS	NS	No, 0.50	High	Low
Park et al,[16] 2020	BMI, kg/m^2	Adults with overweight or obesity	2-3 months	MADF	RD, CER, or TRE	8	307/298	WMD	-0.80 (-1.17 to -0.43)	1 x 10^{-4}	VS	NS, 48.4	NS	NS	No, 0.49	Low	Moderate

© 2021 Patikorn C et al. *JAMA Network Open.*

Park et al,[16] 2020	BMI, kg/m^2	Adults with overweight or obesity	2-6 months	MADF	RD, CER, or TRE	9	374/366	WMD	-0.73 (-1.13 to -0.34)	.001	VS	S, 53.2	NS	NS	No, 0.26	Very low	Moderate
He et al,[19] 2021	Body weight, kg	Adults with overweight or obesity	2-3 months	MADF	CER	2	39/39	WMD	-1.65 (-2.73 to -0.58)	.003	S	NS, 0	NS	NS	N/A	Moderate	Moderate
He et al,[19] 2021	Body weight, kg	Adults with obesity with no comorbidities	2-6 months	MADF	CER	3	73/73	MD	-1.42 (-2.44 to -0.41)	.006	S	NS, 0	NS	NS	No, 0.63	Moderate	Moderate
Park et al,[16] 2020	Body weight, kg	Overweight adults, some with NAFLD	1-3 months	MADF	CER, TRE, or RD	8	307/299	WMD	-1.77 (-3.19 to -0.34)	.02	VS	S, 55.5	S	NS	Yes, 0.07	Very low	Moderate
He et al,[19] 2021	Body weight, kg	Adults with overweight or	3 months	5:2 diet	RD or CER	3	117/173	WMD	-1.67	.003	VS	NS, 0	S	NS	No, 0.68	Very low	Moderate

© 2021 Patikorn C et al. *JAMA Network Open.*

		obesity, some with diabetes							(-2.79 to -0.55)								
Pellegrini et al,[17] 2020	Body weight, kg	Normal weight healthy male adults, some with prediabetes	1-2 months	TRE 12-24 hours	RD or CER	5	44/41	WMD	-0.38 (-0.71 to -0.04)	.03	VS	NS, 0	NS	NS	No, 0.13	Low	Low
He et al,[19] 2021	Fat-free mass, kg	Adults with obesity	2-6 months	MADF	CER	3	73/73	MD	-0.70 (-1.38 to -0.02)	.04	S	NS, 0	NS	NS	No, 0.30	Moderate	Moderate
Cui et al,[13] 2020	Fat-free mass, kg	Healthy adults, some with overweight or obesity	1-12 months	MADF	RD	5	89/73	MD	-1.38 (-2.26 to -0.49)	.002	NS	VS, 91.0	NS	NS	No, 0.50	Low	Low
Park et al,[16] 2020	Fat mass, kg	Adults with overweight or obesity	1-2 months	0-calor	CER or RD	2	39/39	WMD	-1.99	1×10^{-9}	NS	NS, 0	S	NS	N/A	Moderate	Moderate

© 2021 Patikorn C et al. *JAMA Network Open.*

				ie ADF					(-2.59 to -1.38)								
He et al,[19] 2021	Fat mass, kg	Adults with obesity	2-6 months	MADF	CER	3	73/73	MD	-1.05 (-1.98 to -0.13)	.03	VS	NS, 0	NS	NS	No, 0.56	Very low	Moderate
Park et al,[16] 2020	Fat mass, kg	Adults with overweight or obesity, some with NAFLD	2-3 months	MADF	CER, TRE, or RD	5	233/225	WMD	-1.08 (-1.91 to -0.26)	.01	VS	NS, 31.7	S	NS	No, 0.56	Very low	Moderate
Cui et al,[13] 2020	Fat mass, kg	Healthy adults, some with overweight or obesity	1-12 months	MADF	RD	6	119/107	MD	-4.96 (-8.08 to -1.85)	.002	NS	VS, 99.0	S	NS	No, 0.80	Very low	Low
Park et al,[16] 2020	Fat mass, kg	Adults with overweight or obesity, some with NAFLD	2-6 months	MADF	RD, CER, or TRE	6	300/293	WMD	-0.96 (-1.91 to -0.004)	.049	VS	NS, 43.0	S	NS	No, 0.90	Very low	Moderate

© 2021 Patikorn C et al. *JAMA Network Open.*

Moon et al,[15] 2020	Fat mass, kg	Adults with overweight or obesity	2-3 months	TRE 12-21 hours	RD	3	112/96	MD	-2.4 (-2.98 to -1.82)	1×10^{-15}	NS	NS, 0	VS	NS	No, 0.13	Low	Moderate
Lipid profile																	
Meng et al,[14] 2020	LDL-C, mg/dL	Adults with normal weight, overweight, or obesity	3-12 months	MADF	RD with exercise	5	139/140	WMD	-5.14 (-7.44 to -2.83)	1×10^{-5}	VS	NS, 0	S	NS	No, 0.77	Very low	Moderate
Meng et al,[14] 2020	LDL-C, mg/dL	Adults with normal weight, overweight, or obesity	2-12 months	MADF	RD with exercise	7	156/154	WMD	-5.23 (-7.52 to -2.94)	1×10^{-5}	VS	NS, 0	S	NS	No, 0.38	Very low	Moderate
Park et al,[16] 2020	TC, mg/dL	Adults with overweight or obesity, some with NAFLD	2-3 months	MADF	RD, CER, or TRE	5	250/241	WMD	-10.95 (-18.98 to -2.93)	.007	VS	NS, 2.7	S	S	No, 0.75	Very low	Moderate

© 2021 Patikorn C et al. *JAMA Network Open.*

Park et al,[16] 2020	TC, mg/dL	Adults with overweight or obesity, some with NAFLD	2-6 months	MADF	RD or CER	6	317/309	WMD	-8.13 (-15.79 to -0.46)	.04	VS	NS, 19.9	S	S	No, 0.45	Very low	Moderate
Meng et al,[14] 2020	TG, mg/dL	Adults with overweight or obesity with no comorbidities	2 months	MADF	RD or RD with exercise	2	17/14	WMD	-26.84 (-52.33 to -1.35)	.04	S	NS, 0	NS	VS	N/A	Low	Moderate
Park et al,[16] 2020	TG, mg/dL	Adults with overweight or obesity, Some with NAFLD	2-3 months	MADF	RD, CER, or TRE	5	250/241	WMD	-21.67 (-39.44 to -3.89)	.02	VS	NS, 0	S	S	No, 0.33	Very low	Moderate
Glycemic profile																	
Pellegrini et	FPG, mg/dL	Healthy adults, some with overweight,	1-2 months	TRE 12-24	RD or CER	4	57/56	MD	-2.45	.04	S	NS, 0	S	S	No, 0.73	Very low	Low

© 2021 Patikorn C et al. *JAMA Network Open.*

al,[17] 2020		obesity, or chronic disease		hours					(-4.72 to -0.18)								
Pureza et al,[18] 2020	FPG, mg/dL	Overweight adults	4 days to 3 months	TRE 12-21 hours	RD or TRE 12-15 hours	7	148/147	MD	-2.75 (-4.6 to -0.91)	.003	S	VS, 88.7	S	NS	Yes, 0.02	Very low	Moderate
He et al,[19] 2021	Fasting Insulin, mIU/mL	Overweight or obese female adults	3-6 months	5:2 diet	CER	2	90/94	MD	-1.00 (-1.77 to -0.39)	.002	S	NS, 0	NS	NS	N/A	Moderate	Moderate
Pureza et al,[18] 2020	HOMA-IR	Healthy adults with obesity/overweight; some with prediabetes	1 day to 2 months	TRE 18 hours	RD	4	59/60	WMD	-0.51 (-0.82 to -0.19)	.002	VS	S, 50.8	NS	NS	No, 0.56	Very low	Moderate
Others																	

© 2021 Patikorn C et al. *JAMA Network Open.*

Cui et al,[13] 2020	SBP, mmHg	Healthy adults, some with overweight or obesity	1-12 months	MADF	RD	4	90/85	MD	-4.42 (-7.35 to -1.49)	.003	NS	VS, 84.0	S	NS	No, 0.76	Very low	Low
Cui et al,[13] 2020	DBP, mmHg	Healthy adults, some with overweight or obesity	1-12 months	MADF	RD	4	90/85	MD	-3.41 (-5.91 to -0.92)	.003	NS	VS, 80.0	S	NS	No, 0.37	Very low	Low

Abbreviations: 0-calorie ADF: zero-calorie alternate-day fasting; AMSTAR-2: a measurement tool to assess systematic reviews; BMI: body mass index; CER: continuous energy restriction; DBP: diastolic blood pressure; FPG: fasting plasma glucose; HOMA-IR: homeostatic model assessment of insulin resistance; IF: intermittent fasting ;LDL-C: low-density lipoprotein cholesterol; MADF: modified alternate-day fasting; MD: mean difference; NAFLD: nonalcoholic fatty liver disease; N/A: not applicable; RD: regular diet; SBP: systolic blood pressure; TC: total cholesterol; TG: triglycerides; TRE: time restricted feeding; WMD: weighted mean difference

© 2021 Patikorn C et al. *JAMA Network Open.*

eTable 4. Summary of Nonsignificant Effects of Intermittent Fasting and Health Outcomes With Detail of GRADE Assessment

Source	Outcome	Population	Duration of fasting	Type of IF	Control (C)	No. of studies	Sample size (IF/C)	Metric	Random effect size (95% CI)	*P* value	GRADE evidence (Not serious (NS), serious (S), very serious (VS))						AMSTAR-2
											Risk of bias	Inconsistency, I^2, %	Indirectness	Imprecision	publication bias,	Overall certainty of evidence	
Anthropometric measures																	
Cho et al,[11] 2019	BMI, kg/m^2	Adults with overweight or obesity	3 months	MADF	RD or RD with exercise	2	83/83	WMD	-1.00 (-2.96 to 0.96)	.32	VS	NS, 0	NS	NS	N/A	Low	Low
Cho et al,[11] 2019	BMI, kg/m^2	Adults with overweight or obesity	3 months	TRE	RD	3	133/133	WMD	-0.73 (-1.57 to 0.12)	.09	VS	NS, 0	NS	NS	No, 0.28	Low	Low

© 2021 Patikorn C et al. *JAMA Network Open.*

Park et al,[16] 2020	Body weight, kg	Overweight adults, some with NAFLD	1-3 months	MADF	RD, CER, or TRE	9	374/367	WMD	-1.39 (-2.92 to 0.15)	.08	VS	S, 61.0	S	NS	Yes, 0.02	Very low	Moderate
He et al,[19] 2021	Body weight, kg	Adults with overweight or obesity, some with diabetes	6-12 months	5:2 diet	CER	5	307/298	WMD	-0.14 (-1.26 to 0.98)	.81	VS	NS, 27.2	VS	NS	No, 0.83	Very low	Moderate
He et al,[19] 2021	Body weight, kg	Adults with overweight or obesity, some with diabetes	3-24 months	5:2 diet	CER	6	363/358	MD	-0.62 (-1.67 to 0.43)	.25	VS	NS, 36.5	VS	NS	No, 0.68	Very low	Moderate

© 2021 Patikorn C et al. *JAMA Network Open.*

He et al,[19] 2021	Body weight, kg	Adults with overweight or obesity, some with diabetes	3-12 months	5:2 diet	RD or CER	8	424/471	MD	-0.76 (-1.63 to 0.11)	.09	VS	NS, 29.4	S	NS	No, 0.68	Very low	Moderate
Moon et al,[15] 2020	Body weight, kg	Healthy adults, some with overweight or NAFLD	1-3 months	TRE 12-21 hours	CER or RD	6	125/107	WMD	-1.04 (-2.88 to 0.8)	.27	VS	NS, 0	S	NS	No, 0.54	Very low	Moderate
Park et al,[16] 2020	Fat-free mass, kg	Adults with normal weight, overweigh	1-2 months	0-calorie ADF	CER or RD	2	39/39	WMD	-0.67 (-2.39 to 1.05)	.45	S	S, 74.8	S	NS	N/A	Very low	Moderate

© 2021 Patikorn C et al. *JAMA Network Open.*

		t, or obesity														
Park et al,[16] 2020	Fat-free mass, kg	Adults with overweight or obesity, some with NAFLD	3 months	MADF	RD, CER, or TRE	4	214/209	WMD	0.55 (-0.77 to 1.87)	.41	VS	NS, 0	S	NS	No, 0.83	Very low	Moderate
Park et al,[16] 2020	Fat-free mass, kg	Adults with overweight or obesity, some with NAFLD	3-6 months	MADF	RD, CER, or TRE	5	281/277	WMD	0.61 (-0.58 to 1.81)	.32	VS	NS, 0	S	NS	No, 0.85	Very low	Moderate
He et al,[19] 2021	Fat-free mass, kg	Adults with overweigh	3 months	5:2 diet	CER	2	68/72	MD	-0.91	.06	S	NS, 0	S	NS	N/A	Low	Moderate

© 2021 Patikorn C et al. *JAMA Network Open.*

		t or obesity, some with diabetes							(-1.86 to 0.05)							
He et al,[19] 2021	Fat-free mass, kg	Adults with overweight or obesity, some with diabetes	6-12 months	5:2 diet	CER	3	241/228	MD	0.01 (-0.68 to 0.69)	.99	S	NS, 26.1	S	NS	No, 0.23	Low	Moderate
He et al,[19] 2021	Fat-free mass, kg	Adults with overweight or obesity, some with diabetes	3-12 months	5:2 diet	CER	5	309/300	MD	-0.31 (-1.35 to 0.72)	.55	VS	NS, 16.7	VS	NS	No, 0.48	Very low	Moderate

© 2021 Patikorn C et al. *JAMA Network Open.*

Pellegrini et al,[17] 2020	Fat-free mass, kg	Healthy adults, some with active lifestyle or normal body habitus	1-2 months	TRE 12-24 hours	RD or CER	4	44/41	WMD	0.00 (-0.78 to 0.79)	.99	VS	NS, 0	NS	NS	No, 0.77	Low	Low
Moon et al,[15] 2020	Fat-free mass, kg	Normal weight, overweight, or obese young adults, some with NAFLD	2-3 months	TRE 12-21 hours	RD or CER	5	132/116	MD	-0.29 (-1.25 to 0.68)	.56	NS	NS, 0	S	NS	No, 0.88	Moderate	Moderate

© 2021 Patikorn C et al. *JAMA Network Open.*

He et al,[19] 2021	Fat mass, kg	Adults with overweight or obesity, some with diabetes	3 months	5:2 diet	CER	2	68/72	MD	-0.4 (-2.13 to 1.33)	.65	VS	NS, 23.6	S	NS	N/A	Very low	Moderate
He et al,[19] 2021	Fat mass, kg	Adults with overweight or obesity, some with diabetes	6-12 months	5:2 diet	CER	3	241/228	MD	-0.29 (-1.95 to 1.38)	.74	VS	NS, 42.7	VS	NS	No, 0.96	Very low	Moderate
He et al,[19] 2021	Fat mass, kg	Adults with overweight or obesity,	3-12 months	5:2 diet	CER	5	309/300	MD	-0.31 (-1.35 to 0.72)	.55	VS	NS, 16.7	S	NS	No, 0.48	Very low	Moderate

© 2021 Patikorn C et al. *JAMA Network Open.*

		some with diabetes														
Cioffi et al,[9] 2018	Fat mass, kg	Overweight adults with no comorbidities	2-6 months	5:2 diet	CER	4	174/138	WMD	-1.01 (-2.9 to 0.88)	.30	VS	NS, 0	S	NS	No, 0.26	Very low	Moderate
Pellegrini et al,[17] 2020	Fat mass, kg	Healthy adults, some with active lifestyle or normal body habitus	2 months	TRE 12-24 hours	RD	4	44/41	WMD	-0.83 (-1.89 to 0.24)	.13	VS	NS, 31.0	NS	NS	No, 0.22	Low	Low
Roman et al,[12] 2019	Hip circumference, cm	Adults with overweigh	4-6 months	5:2 diet	CER	2	128/94	MD	-1.45 (-7.54 to 4.64)	.64	VS	NS, 0	NS	S	N/A	Very low	Low

© 2021 Patikorn C et al. *JAMA Network Open.*

		t or obesity															
Park et al,[16] 2020	Waist circumference, cm	Adults with overweight or obesity, some with NAFLD	2-3 months	MADF	RD, CER, or TRE	5	249/244	WMD	-1.50 (-3.08 to 0.08)	.06	VS	S, 50.8	S	NS	No, 0.63	Very low	Moderate
He et al,[19] 2021	Waist circumference, cm	Adults with overweight or obesity	3-6 months	5:2 diet	CER	4	156/164	MD	-0.53 (-2.56 to 1.49)	.61	VS	NS, 27.4	S	NS	No, 0.29	Very low	Moderate
Cioffi et al,[9] 2018	Waist circumference, cm	Adults with overweight or obesity,	2-6 months	5:2 diet	CER	5	209/176	WMD	-0.17 (-1.74 to 1.39)	.83	VS	NS, 5.6	S	NS	No, 0.23	Very low	Moderate

© 2021 Patikorn C et al. *JAMA Network Open.*

		some with metabolic syndrome														
He et al,[19] 2021	Waist circumference, cm	Adults with overweight or obesity, some with diabetes	3-12 months	5:2 diet	CER	5	309/300	MD	-0.28 (-0.91 to 0.35)	.38	VS	NS, 34.1	S	NS	No, 0.25	Very low	Moderate
Lipid profile																	
Meng et al,[14] 2020	HDL-C, mg/dL	Adults with overweight or obesity	2 months	MADF	RD or RD with exercise	2	17/14	WMD	-4.28 (-10.15 to 1.59)	.15	S	NS, 0	NS	S	N/A	Low	Moderate
Meng et al,[14] 2020	HDL-C, mg/dL	Adults with overweigh	3-12 months	MADF	RD or RD with	5	139/140	WMD	-0.93	.70	VS	S, 71.8	NS	S	No, 0.57	Very low	Moderate

© 2021 Patikorn C et al. *JAMA Network Open.*

		t or obesity			exercise				(-5.63 to 3.77)								
Meng et al,[14] 2020	HDL-C, mg/dL	Adults with overweight or obesity	2-12 months	MADF	RD or RD with exercise	7	156/154	WMD	-1.71 (-5.58 to 2.16)	.39	VS	S, 64.8	NS	NS	No, 0.29	Very low	Moderate
Park et al,[16] 2020	HDL-C, mg/dL	Adults with overweight or obesity, some with NAFLD	2-6 months	MADF	RD or CER	6	317/309	WMD	-0.89 (-4.23 to 2.46)	.60	VS	S, 61.9	NS	NS	No, 0.55	Low	Moderate
Park et al,[16] 2020	HDL-C, mg/dL	Adults with overweight or	2-3 months	MADF	RD, CER, or TRE	5	250/241	WMD	0.22 (-3.14 to 3.59)	.90	VS	NS, 44.7	S	NS	Yes, 0.03	Very low	Moderate

© 2021 Patikorn C et al. *JAMA Network Open.*

		obesity, some with NAFLD															
Meng et al,[14] 2020	HDL-C, mg/dL	Adults with overweight or obesity	2-12 months	5:2 diet	RD or CER	3	40/39	WMD	2.09 (-3.41 to 7.6)	.46	VS	S, 52.3	NS	NS	No, 0.21	Very low	Moderate
Cioffi et al,[9] 2018	HDL-C, mg/dL	Adults with overweight or obesity, some with metabolic syndrome	2-6 months	5:2 diet	CER	5	209/176	MD	0.60 (-1.14 to 2.34)	.50	VS	NS, 0	NS	NS	No, 0.31	Low	Moderate
Pellegrini	HDL-C, mg/dL	Healthy adults, some with	1-2 months	TRE 12-24	RD	4	34/33	WMD	9.14	.16	VS	VS, 81.0	NS	VS	No, 0.27	Very low	Low

© 2021 Patikorn C et al. *JAMA Network Open.*

et al,[17] 2020		obesity or prediabetes		hours					(-3.69 to 21.97)								
Meng et al,[14] 2020	LDL-C, mg/dL	Adults with normal weight, overweight, or obesity	2 months	MADF	RD with exercise	2	17/14	WMD	-11.95 (-31.35 to 7.45)	.23	S	NS, 0	NS	S	N/A	Low	Moderate
Cioffi et al,[9] 2018	LDL-C, mg/dL	Adults with overweight or obesity	2-6 months	MADF	CER	2	49/49	MD	2.96 (-3.11 to 9.03)	.34	S	S, 53.4	NS	S	N/A	Low	Moderate
Cioffi et al,[9] 2018	LDL-C, mg/dL	Adults with overweight or	2-6 months	5:2 diet	CER	6	189/194	MD	1.41 (-0.64 to 3.45)	.18	S	NS, 18.5	S	NS	No, 0.15	Low	Moderate

© 2021 Patikorn C et al. *JAMA Network Open.*

		obesity, some with metabolic syndrome or diabetes															
Park et al,[16] 2020	LDL-C, mg/dL	Adults with overweight or obesity	2-3 months	MADF	RD, CER, or TRE	6	261/250	WMD	0.62 (-6.13 to 7.38)	.86	VS	NS, 36.7	NS	S	Yes, 0.08	Very low	Moderate
Park et al,[16] 2020	LDL-C, mg/dL	Adults with overweight or obesity	2-6 months	MADF	RD, CER, or TRE	7	328/318	WMD	0.88 (-4.49 to 6.25)	.75	VS	NS, 25.1	NS	S	Yes, 0.07	Very low	Moderate
Pellegrini	LDL-C, mg/dL	Adults with normal	1-2 months	TRE 12-24	RD or CER	4	57/56	MD	1.36	.34	VS	S, 53.9	S	S	No, 0.27	Very low	Low

© 2021 Patikorn C et al. *JAMA Network Open.*

et al,[17] 2020		weight, overweight, or obesity		hours					(-1.43 to 4.14)								
Pureza et al,[18] 2020	LDL-C, mg/dL	Adults with obesity	4 days to 3 months	TRE 12-21 hours	RD or TRE 12-15 hours	4	97/96	WMD	0.38 (-2.97 to 3.73)	.82	S	S, 73.8	S	NS	No, 0.96	Very low	Moderate
Meng et al,[14] 2020	TC, mg/dL	Adults with overweight or obesity	2 months	MADF	RD or RD with exercise	2	17/14	WMD	-14.37 (-36.12 to 7.38)	.20	S	NS, 21.9	NS	S	N/A	Low	Moderate
Meng et al,[14] 2020	TC, mg/dL	Adults with overweight or obesity	3-4 months	MADF	RD or CER with exercise	5	139/140	WMD	-3.41 (-10.44 to 3.62)	.34	VS	S, 61.5	NS	S	No, 0.38	Very low	Moderate

© 2021 Patikorn C et al. *JAMA Network Open.*

Meng et al,[14] 2020	TC, mg/dL	Adults with overweight or obesity	2-4 months	MADF	RD, RD with exercise, or CER with exercise	7	156/154	WMD	-4.50 (-10.94 to 1.95)	.17	VS	S, 51.1	NS	S	No, 0.69	Very low	Moderate
Meng et al,[14] 2020	TC, mg/dL	Adults with overweight or obesity	2-12 months	5:2 diet	RD	3	40/39	WMD	-6.41 (-15.80 to 2.99)	.18	VS	NS, 0	NS	S	No, 0.76	Very low	Moderate
Cioffi et al,[9] 2018	TC, mg/dL	Adults with overweight or obesity,	2-6 months	5:2 diet	CER	5	209/176	MD	0.11 (-4.85 to 5.07)	.97	VS	NS, 0	NS	NS	No, 0.63	Very low	Moderate

© 2021 Patikorn C et al. *JAMA Network Open.*

		some with metabolic syndrome														
Pureza et al,[18] 2020	TC, mg/dL	Adults with overweight or obesity, some with prediabetes or diabetes	4 days to 3 months	TRE 12-21 hours	RD or TRE 12-15 hours	3	33/32	WMD	6.34 (-3.45 to 16.12)	.21	VS	VS, 82.6	S	S	Yes, 0.01	Very low	Moderate
Pellegrini et al,[17] 2020	TC, mg/dL	Healthy adults, some with obesity and prediabetes	1-2 months	TRE 12-24 hours	RD	4	34/33	WMD	9.14 (-3.69 to 21.97)	.16	VS	VS, 81	NS	S	No, 0.27	Very low	Low

© 2021 Patikorn C et al. *JAMA Network Open.*

Meng et al,[14] 2020	TG, mg/dL	Adults with overweight or obesity with no comorbidities	2-12 months	MADF	RD or CER	5	139/140	WMD	-2.88 (-15.85 to 10.09)	.66	VS	S, 67.0	NS	S	No, 0.55	Very low	Moderate
Meng et al,[14] 2020	TG, mg/dL	Adults with overweight or obesity with no comorbidities	2-4 months	MADF	RD, or RD with exercise, or CER with exercise	7	156/154	WMD	-2.42 (-14.94 to 10.09)	.70	VS	S, 56.7	NS	S	No. 0.48	Very low	Moderate

© 2021 Patikorn C et al. *JAMA Network Open.*

Park et al,[16] 2020	TG, mg/dL	Adults with overweight or obesity, some with NAFLD	2-6 months	MADF	RD or CER	6	317/309	WMD	-9.98 (-26.01 to 6.06)	.22	VS	NS, 25.2	NS	S	Yes, 0.06	Very low	Moderate
Cioffi et al,[9] 2018	TG, mg/dL	Adults with overweight or obesity, some with metabolic syndrome	2-6 months	5:2 diet	CER	5	209/176	MD	0.05 (-5.45 to 5.54)	.99	VS	NS, 0	NS	S	No, 0.96	Very low	Moderate
Pureza et al,[18] 2020	TG, mg/dL	Adults with overweight or	4 days to 3 months	TRE 12-21	RD or TRE 12-15 hours	6	68/65	WMD	1.63 (-7.84 to 11.09)	.74	VS	VS, 80.0	NS	S	No, 0.58	Very low	Moderate

© 2021 Patikorn C et al. *JAMA Network Open.*

		obesity, some with prediabetes or diabetes		hours													
Pellegrini et al,[17] 2020	TG, mg/dL	Healthy adults, some with obesity and prediabetes	1-2 months	TRE 12-24 hours	RD	4	34/33	WMD	6.24 (-13.4 to 25.88)	.53	VS	VS, 86.8	NS	S	No, 0.34	Very low	Low
Glycemic profile																	
Cui et al,[13] 2020	FPG, mg/dL	Adults with normal weight, overweight, or	2-12 months	MADF	RD	4	88/56	MD	-3.02 (-6.52 to 0.48)	.09	NS	VS, 89.0	NS	S	No, 0.46	Very low	Low

© 2021 Patikorn C et al. *JAMA Network Open.*

		obesity, some with NAFLD															
Park et al,[16] 2020	FPG, mg/dL	Adults with overweight or obesity	2-3 months	MADF	RD, CER, or TRE	6	261/250	WMD	-1.11 (-5.57 to 3.37)	.63	VS	S, 63.0	NS	NS	No, 0.51	Very low	Moderate
Park et al,[16] 2020	FPG, mg/dL	Adults with overweight or obesity	2-6 months	MADF	RD, CER, or TRE	7	328/318	WMD	-0.61 (-3.76 to 2.53)	.70	VS	S, 56.1	NS	NS	No, 0.49	Very low	Moderate
He et al,[19] 2021	HbA1c, %	Adults with overweight or obesity,	3-12 months	5:2 diet	CER	4	192/200	WMD	0.00 (-0.08 to 0.07)	.93	VS	NS, 0	S	NS	No, 0.66	Very low	Moderate

© 2021 Patikorn C et al. *JAMA Network Open.*

		some with diabetes															
Park et al,[16] 2020	Fasting Insulin, mIU/mL	Adults with overweight or obesity	2-3 months	MADF	RD or CER	3	70/67	WMD	-2.01 (-4.55 to 0.53)	.12	VS	NS, 0	NS	NS	No, 0.41	Low	Moderate
Park et al,[16] 2020	Fasting Insulin, mIU/mL	Adults with overweight or obesity	2-6 months	MADF	RD or CER	4	137/135	WMD	-0.19 (-2.32 to 1.94)	.86	VS	NS, 30.9	S	NS	No, 0.54	Very low	Moderate
Pureza et al,[18] 2020	Fasting Insulin, mIU/mL	Adults with obesity	4 days to 3 months	TRE 12-21 hours	RD or TRE 12-15 hours	5	48/46	WMD	-1.1 (-2.85 to 0.65)	.22	VS	VS, 75.3	VS	NS	No, 0.30	Very low	Moderate

© 2021 Patikorn C et al. *JAMA Network Open.*

Pellegrini et al,[17] 2020	Fasting Insulin, mIU/mL	Healthy adults, some with obesity and prediabetes	1-2 months	TRE 12-24 hours	RD	3	34/33	WMD	-0.69 (-1.64 to 0.25)	.15	VS	NS, 48.8	S	NS	No, 0.76	Very low	Low
Park et al,[16] 2020	HOMA-IR	Adults with overweight or obesity	2-3 months	MADF	RD or CER	3	70/67	WMD	-0.48 (-1.18 to 0.22)	.18	VS	NS, 0	NS	NS	No, 0.16	Very low	Moderate
He et al,[19] 2021	HOMA-IR	Overweight or obese female adults	3-6 months	5:2 diet	CER	2	90/94	MD	-0.23 (-0.52 to 0.06)	.12	VS	S, 61.2	NS	NS	N/A	Very low	Moderate

© 2021 Patikorn C et al. *JAMA Network Open.*

Pellegrini et al,[17] 2020	HOMA-IR	Adults with normal weight, overweight, or obesity, some with prediabetes	1 month	TRE 12-24 hours	RD	2	17/16	WMD	-0.33 (-1.14 to 0.47)	.42	VS	S, 71.4	NS	NS	N/A	Low	Low
Blood pressure																	
Park et al,[16] 2020	SBP, mmHg	Adults with overweight or obesity	2-6 months	MADF	RD, or CER	4	146/147	WMD	-4.34 (-9.94 to 1.26)	.13	VS	S, 71.2	NS	NS	No, 0.45	Very low	Moderate
Harris et al,[10] 2018	SBP, mmHg	Adults with overweight	3-6 months	5:2 diet	RD or CER	2	90/80	WMD	-4.29 (-11.13	.22	S	S, 53.1	S	S	N/A	Very low	Moderate

© 2021 Patikorn C et al. *JAMA Network Open.*

		t or obesity							to 2.56)							
Cioffi et al,[9] 2018	SBP, mmHg	Adults with overweight or obesity, some with metabolic syndrome or diabetes	2-6 months	5:2 diet	CER	5	171/176	MD	-0.44 (-5.96 to 5.07)	.86	VS	VS, 79.6	S	S	No, 0.56	Very low	Moderate
Pellegrini et al,[17] 2020	SBP, mmHg	Adults with normal weight, overweight, or obesity,	1-2 months	TRE 12-24 hours	CER	2	23/23	MD	-2.27 (-19.52 to 14.98)	.80	VS	VS, 88.9	S	S	N/A	Very low	Low

© 2021 Patikorn C et al. *JAMA Network Open.*

		some with prediabetes															
Park et al,[16] 2020	DBP, mmHg	Adults with overweight or obesity	2-6 months	MADF	RD, or CER	4	146/147	WMD	-0.97 (-2.61 to 0.67)	.25	VS	NS, 0	NS	NS	No, 0.55	Low	Moderate
Harris et al,[10] 2018	DBP, mmHg	Adults with overweight or obesity	3-6 months	5:2 diet	RD or CER	2	90/80	WMD	-3.81 (-11.64 to 4.02)	.34	S	S, 64.1	S	S	N/A	Very low	Moderate
Cioffi et al,[9] 2018	DBP, mmHg	Adults with overweight or obesity, some with	2-6 months	5:2 diet	CER	5	134/136	MD	0.22 (-1.69 to 2.12)	.83	VS	NS, 0	NS	NS	No, 0.60	Low	Moderate

© 2021 Patikorn C et al. *JAMA Network Open.*

		metabolic syndrome or diabetes															
Pellegrini et al,[17] 2020	DBP, mmHg	Adults with normal weight, overweight, or obesity, some with prediabetes	1-2 months	TRE 12-24 hours	CER	2	23/23	MD	-2.76 (-16.27 to 10.75)	.69	VS	VS, 88.1	S	S	N/A	Very low	Low
Others																	
Pureza et al,[18] 2020	Ghrelin, pg/mL	Normal weight, or overweight adults,	4 days to 1 month	TRE 12-21	RD or TRE 12-15 hours	3	38/38	WMD	-18.46 (-45.94	.19	VS	VS, 91.8	S	S	No, 0.18	Very low	Moderate

© 2021 Patikorn C et al. *JAMA Network Open.*

		some prediabetes		hours					to 9.03)								
Cho et al,[11] 2019	Adiponectin, ng/mL	Adults with overweight or obesity	3-6 months	MADF	CR, RD	3	188/188	WMD	772.2 (-270.16 to 1814.57)	.15	VS	NS, 0	NS	VS	No, 0.75	Very low	Low
Cho et al,[11] 2019	Leptin, ng/mL	Adults with overweight or obesity	3-6 months	MADF	CR, RD	3	188/188	WMD	-2.13 (-11.62 to 7.35)	.66	VS	NS, 0	NS	S	No, 0.97	Very low	Low
Park et al,[16] 2020	CRP, mg/L	Adults with overweight or obesity	2-6 months	MADF	RD or diet	3	102/100	WMD	-0.16 (-0.53 to 0.21)	.40	VS	S, 54.2	NS	NS	Yes, 0.01	Very low	Moderate

© 2021 Patikorn C et al. *JAMA Network Open.*

Abbreviations: 0-calorie ADF: zero-calorie alternate-day fasting; AMSTAR-2: a measurement tool to assess systematic reviews; BMI: body mass index; CER: continuous energy restriction; CRP: C-reactive protein; DBP: diastolic blood pressure; FPG: fasting plasma glucose; HOMA-IR: homeostatic model assessment of insulin resistance; IF: intermittent fasting ;LDL-C: low-density lipoprotein cholesterol; MADF: modified alternate-day fasting; MD: mean difference; NAFLD: nonalcoholic fatty liver disease; N/A: not applicable; RD: regular diet; SBP: systolic blood pressure; TC: total cholesterol; TG: triglycerides; TRE: time restricted eating; WMD: weighted mean difference

© 2021 Patikorn C et al. *JAMA Network Open.*

eTable 5. Summary of Sensitivity Analyses

Outcome	Population	Duration of fasting	Type of IF	Control	Metric	Primary analysis			Sensitivity analysis: Excluding studies with high risk of bias			Sensitivity analysis: Excluding studies with small sample size (25th percentile)		
						**No. of studies**	**Effect size (95% CI)**	**GRADE**	**No. of studies**	**Effect size (95% CI)**	**GRADE**	**No. of studies**	**Effect size (95% CI)**	**GRADE**
Anthropometric measures														
BMI, kg/m² in Cui et al,[23] 2020	Healthy adults, some with overweight, obesity, or NAFLD	1-2 months	MADF	RD	MD	4	-1.20 (-1.44 to -0.96)	High	N/A – remaining studies are not enough to conduct meta-analysis			3	-1.20 (-1.45 to -0.96)	High
BMI, kg/m² in Park	Adults with overweight or obesity	2-3 months	MADF	RD, CER, or TRE	WMD	8	-0.80 (-1.17 to	Low	4	-0.79 (-1.29 to	Low	6	-0.83 (-1.24 to	Very low

© 2021 Patikorn C et al. *JAMA Network Open.*

et al,[26] 2020							-0.43)			-0.30)			-0.43)	
BMI, kg/m^2 in Park et al,[26] 2020	Adults with overweight or obesity	2-6 months	MADF	RD, CER, or TRE	WMD	9	-0.73 (-1.13 to -0.34)	Very low	4	-0.79 (-1.29 to -0.30)	Low	7	-0.8 (-1.19 to -0.41)	Very low
Body weight, kg in He et al,[25] 2021	Adults with overweight or obesity	2-3 months	MADF	CER	WMD	2	-1.65 (-2.73 to -0.58)	Moderate	N/A – remaining studies are not enough to conduct meta-analysis			N/A – remaining studies are not enough to conduct meta-analysis		
Body weight, kg in He et al,[25] 2021	Adults with obesity with no comorbidities	2-6 months	MADF	CER	MD	3	-1.42 (-2.44 to -0.41)	Moderate	N/A – remaining studies are not enough to conduct meta-analysis			2	-1.65 (-2.73 to -0.58)	Moderate

© 2021 Patikorn C et al. *JAMA Network Open.*

Body weight, kg in Park et al,[26] 2020	Overweight adults, some with NAFLD	1-3 months	MADF	RD, CER, or TRE	WMD	8	-1.77 (-3.19 to -0.34)	Very low	3	-2.55 (-4.43 to -0.68)	Moderate	6	-1.84 (-3.36 to -0.33)	Very low
Body weight, kg in He et al,[25] 2021	Adults with overweight or obesity, some with diabetes	3 months	5:2 diet	RD or CER	WMD	3	-1.67 (-2.79 to -0.55)	Very low	N/A – remaining studies are not enough to conduct meta-analysis			N/A – remaining studies are not enough to conduct meta-analysis		
Body weight, kg in Pellegrini et al,[24] 2020	Normal weight healthy male adults, some with prediabetes	1-2 months	TRE 12-24 hours	RD or CER	WMD	5	-0.38 (-0.71 to -0.04)	Low	N/A – remaining studies are not enough to conduct meta-analysis			3	-0.40 (-0.78 to -0.01)	Low

© 2021 Patikorn C et al. *JAMA Network Open.*

Fat-free mass, kg in He et al,[25] 2021	Adults with obesity	2-6 months	MADF	CER	MD	3	-0.70 (-1.38 to -0.02)	Moderate	N/A – remaining studies are not enough to conduct meta-analysis	2	-0.8 (-1.51 to -0.09)	Moderate
Fat-free mass, kg in Cui et al,[23] 2020	Healthy adults, some with overweight or obesity	1-12 months	MADF	RD	MD	5	-1.38 (-2.26 to -0.49)	Low	N/A – remaining studies are not enough to conduct meta-analysis	4	-1.08 (-1.99 to -0.17)	Low
Fat mass, kg in Park et al,[26] 2020	Adults with overweight or obesity	1-2 months	0-calorie ADF	RD or CER	WMD	2	-1.99 (-2.59 to -1.38)	Moderate	N/A – remaining studies are not enough to conduct meta-analysis	N/A – remaining studies are not enough to conduct meta-analysis		
Fat mass, kg in He	Adults with obesity	2-6 months	MADF	CER	MD	3	-1.05 (-1.98 to -0.13)	Very low	N/A – remaining studies are not enough to conduct meta-analysis	2	-1.23 (-2.2 to -0.26)	Low

© 2021 Patikorn C et al. *JAMA Network Open.*

et al,[25] 2021												
Fat mass, kg in Park et al,[26] 2020	Adults with overweight or obesity, some with NAFLD	2-3 months	MADF	RD, CER, or TRE	WMD	5	-1.08 (-1.91 to -0.26)	Very low	N/A – remaining studies are not enough to conduct meta-analysis	4	-1.17 (-2.04 to - 0.29)	Very low
Fat mass, kg in Cui et al,[23] 2020	Healthy adults, some with overweight or obesity	1-12 months	MADF	RD	MD	6	-4.96 (-8.08 to -1.85)	Very low	N/A – remaining studies are not enough to conduct meta-analysis	5	-5.68 (-9.15 to -2.21)	Low
Fat mass, kg in Park et al,[26] 2020	Adults with overweight or obesity, some with NAFLD	2-6 months	MADF	RD, CER, or TRE	WMD	6	-0.96 (-1.91 to - 0.004)	Very low	N/A – remaining studies are not enough to conduct meta-analysis	5	-1.03 (-2.04 to -0.03)	Very low

© 2021 Patikorn C et al. *JAMA Network Open.*

Fat mass, kg in Moon et al,[4] 2020	Adults with overweight or obesity	2-3 months	TRE 12-20 hours	RD	MD	3	-2.40 (-2.98 to -1.82)	Low	N/A – remaining studies are not enough to conduct meta-analysis			2	-2.35 (-3.22 to -1.48)	Low
Lipid profile														
LDL-C, mg/dL in Meng et al,[6] 2020	Adults with normal weight, overweight, or obesity	3-12 months	MADF	RD with exercise	WMD	5	-5.14 (-7.44 to -2.83)	Very low	2	-3.33 (-11.93 to 5.27)	Moderate	4	-5.17 (-7.50 to -2.83)	Low
LDL-C, mg/dL in Meng et al,[6] 2020	Adults with normal weight, overweight, or obesity	2-12 months	MADF	RD with exercise	WMD	7	-5.23 (-7.52 to -2.94)	Very low	4	-4.75 (-12.6 to 3.11)	Low	5	-5.14 (-7.44 to -2.83)	Low
TC, mg/dL in Park	Adults with overweight or obesity,	2-3 months	MADF	RD, CER, or TRE	WMD	5	-10.95	Very low	N/A – remaining studies are not enough to conduct meta-analysis			4	-9.77 (-17.92	Low

© 2021 Patikorn C et al. *JAMA Network Open.*

et al,[26] 2020	some with NAFLD						(-18.98 to -2.93)						to -1.63)	
TC, mg/dL in Park et al,[26] 2020	Adults with overweight or obesity, some with NAFLD	2-6 months	MADF	RD or CER	WMD	6	-8.13 (-15.79 to -0.46)	Very low	2	-0.8 (-10.41 to 8.81)	Very low	5	-6.89 (-14.24 to 0.46)	Very low
TG, mg/dL in Meng et al,[6] 2020	Adults with overweight or obesity with no comorbidities	2 months	MADF	RD or RD with exercise	WMD	2	-26.84 (-52.33 to -1.35)	Low	N/A – remaining studies are not enough to conduct meta-analysis			N/A – remaining studies are not enough to conduct meta-analysis		
TG, mg/dL in Park	Adults with overweight or obesity,	2-3 months	MADF	RD, CER, or TRE	WMD	5	-21.67 (-39.44	Very low	N/A – remaining studies are not enough to conduct meta-analysis			4	-21.16 (-39.18	Very low

© 2021 Patikorn C et al. *JAMA Network Open.*

et al,[26] 2020	Some with NAFLD						to - 3.89)						to - 3.15)	
Glycemic profile														
FPG, mg/dL in Pellegrini et al,[24] 2020	Healthy adults, some with overweight, obesity, or chronic disease	1-2 months	TRE 12-24 hours	RD or CER	MD	4	-2.45 (-4.72 to -0.18)	Very low	N/A – remaining studies are not enough to conduct meta-analysis			3	-2.45 (-4.72 to -0.18)	Very low
FPG, mg/dL in Pureza et al,[7] 2020	Overweight adults	4 days to 3 months	TRE 12-21 hours	RD or TRE 12-15 hours	MD	7	-2.75 (-4.6 to -0.91)	Very low	4	-2.87 (-5.46 to -0.28)	Very low	5	-2.51 (-4.54 to -0.47)	Very low
Fasting insulin,	Overweight or obese	3-6 months	5:2 diet	CER	MD	2	-1.00 (-1.77 to	Moderat e	N/A – remaining studies are not enough to conduct meta-analysis			N/A – remaining studies are not enough to conduct meta-analysis		

© 2021 Patikorn C et al. *JAMA Network Open.*

mIU/mL in He et al,[25] 2021	female adults						-0.39)					
HOMA-IR in Pureza et al,[7] 2020	Healthy adults with overweight or obesity; some with prediabetes	1 day to 2 months	TRE 18 hours	RD	WMD	4	-0.51 (-0.82 to -0.19)	Very low	N/A – remaining studies are not enough to conduct meta-analysis	3	-0.36 (-0.69 to -0.02)	Very low
Others												
SBP, mmHg in Cui et al,[23] 2020	Healthy adults, some with overweight or obesity	1-12 months	MADF	RD	MD	4	-4.42 (-7.35 to -1.49)	Very low	N/A – remaining studies are not enough to conduct meta-analysis	3	-4.84 (-8.09 to -1.60)	Low
DBP, mmHg in Cui	Healthy adults, some with	1-12 months	MADF	RD	MD	4	-3.41 (-5.91 to	Very low	N/A – remaining studies are not enough to conduct meta-analysis	3	-3.49 (-6.33 to	Low

© 2021 Patikorn C et al. *JAMA Network Open.*

et al,[23] 2020	overweight or obesity						-0.92)				-0.65)	

Abbreviations: 0-calorie ADF: zero-calorie alternate-day fasting; BMI: body mass index; CER: continuous energy restriction; DBP: diastolic blood pressure; FPG: fasting plasma glucose; HOMA-IR: homeostatic model assessment of insulin resistance; IF: intermittent fasting ;LDL-C: low-density lipoprotein cholesterol; MADF: modified alternate-day fasting; MD: mean difference; NAFLD: nonalcoholic fatty liver disease; RCTs: randomized controlled trials; RD: regular diet; SBP: systolic blood pressure; TC: total cholesterol; TG: triglycerides; TRE: time restricted eating; WMD: weighted mean difference

© 2021 Patikorn C et al. *JAMA Network Open.*
